# Engineered Coiled-Coils Convert Cholera Toxin B‑Pentamers into Programmable Membrane Fusogens

**DOI:** 10.1021/acsnano.6c01578

**Published:** 2026-05-13

**Authors:** Wenyue Dai, Erik Kempmann, Francesca Rosato, Maria Nikolova, Lina Siukstaite, Tomasz P. Kamiński, Andrew Booth, Maryam S. K. Ishmael, Chunyue Wang, George R. Heath, Paul A. Beales, Ralf P. Richter, Winfried Römer, Michael E. Webb, W. Bruce Turnbull

**Affiliations:** † School of Chemistry, 4468 University of Leeds, Leeds LS2 9JT, U.K.; ‡ Astbury Centre for Structural Molecular Biology, 4468 University of Leeds Leeds LS2 9JT, U.K.; § Faculty of Biology, Synthetic Biology of Signalling Processes Lab, 5894 University of Freiburg, Freiburg 79104, Germany; ∥ Signalling Research Centres BIOSS and CIBSS, University of Freiburg, Freiburg 79104, Germany; ⊥ School of Biomedical Sciences, University of Leeds Leeds LS2 9JT, U.K.; # School of Physics and Astronomy, University of Leeds Leeds LS2 9JT, U.K.; ∇ Bragg Centre for Materials Research, University of Leeds Leeds LS2 9JT, U.K.

**Keywords:** membrane fusion, programmable biointerfaces, coiled-coil protein engineering, lectin−glycolipid interactions, synthetic fusogens, nanobiotechnology

## Abstract

Membrane fusion is central to biological function and bioengineering, yet few design rules exist that enable proteins to be programmed to drive fusion at defined membrane interfaces. Here, we show that cholera toxin B-subunit (CTB), a naturally occurring glycolipid-binding pentamer, can be re-engineered into a programmable membrane fusogen by assembling two CTB units through rationally designed coiled-coil linkers attached to the CTA2 peptide that threads through the CTB pentamer. Using discrete parallel and antiparallel coiled-coil architectures, we generated CTB dimers with defined orientations and examined their ability to drive fusion of giant unilamellar vesicles containing the CTB ligand ganglioside GM1. Fusion efficiency was evaluated using a fluorescence resonance energy transfer (FRET)-based lipid mixing assay, while flow cytometry, confocal microscopy, and quartz crystal microbalance with dissipation monitoring (QCM-D) provided mechanistic insights. Both parallel and antiparallel CTB dimers induced cross-linking and full fusion; strikingly, fusogenic efficiency was governed primarily by the length of the CTA2 linker rather than coiled-coil orientation. These findings establish a generalizable strategy for engineering lectin-based fusogens with tunable activity, defining linker geometry as a key design parameter and advancing the development of programmable membrane fusion platforms for drug delivery and synthetic cell systems.

## Graphical abstract

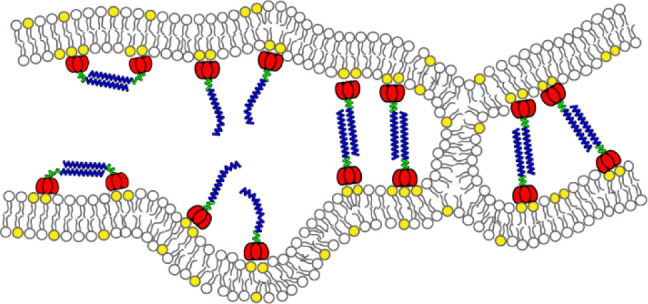

Membrane fusion is a critical process in a range of physiological activities including fertilization,^1^ synaptic transmission,^2^ vesicle trafficking, and viral invasion.^3,4^ However, it requires overcoming of an energy barrier and disrupting the hydration shell between opposing bilayers, which is not a spontaneous process. Natural proteins have been well-documented to facilitate vesicle fusion, most prominently SNAREs (soluble *N*-ethylmaleimide-sensitive factor activating protein receptors).^5,6^ SNAREs are based on coiled-coil motifs that have hydrophobic interfaces between α-helices, with so-called knob-into-hole packing, and are stabilized by flanking salt bridges.^7^ The canonical example of a SNARE complex comprises a four helix bundle of three polypeptides: syntaxin-1A and synaptobrevin, which are integral membrane proteins, and SNAP-25, which is associated with the membrane by palmitoylation. When the procoils embedded in the vesicle and target membranes form a coiled-coil assembly, stress induced in the membranes promotes their fusion.^8^

Artificial constructs that mediate membrane fusion have been developed based on a variety of molecular recognition systems including boronic acid/cis-diols,^9^ vancomycin/d-Ala-d-Ala,^10^ membrane-anchored peptides,^11^ and complementary DNA strands.^12–14^ Non-natural lipidated coiled-coils, in particular, have been studied extensively for their membrane fusion properties.^15–22^ Factors that affect fusion efficiency include the stability of the coiled-coil,^16^ the length of linker between the lipid and the procoil,^17^ coiled-coil orientation,^18^ and additional interactions between the procoil and membrane.^19^

Recently, we reported that multimeric assemblies of the carbohydrate-binding cholera toxin B-subunit (CTB) can induce membrane fusion.^23^ Cholera toxin is an AB_5_ complex consisting of a catalytic A1 subunit (CTA1) connected by a disulfide bond to a linker A2 peptide (CTA2), which passes through the central pore of the pentameric CTB (Figure 1a).^24^ The nontoxic CTB subunit is a lectin that binds with high affinity (40 nM per site) to up to five ganglioside GM1 molecules.^25^ Binding drives GM1 clustering,^26^ generating negative membrane curvature and tubular invaginations, which initiate endocytosis.^27,28^ However, a single CTB pentamer cannot cross-link or fuse membranes because its GM1-binding sites are orientated in a single direction.^29^ In our earlier work, we conferred fusogenic activity on CTB by creating Strep–(AB_5_)_*n*_ assemblies, in which biotinylation of the CTA2 peptide allowed CTA2/CTB units to be cross-linked using streptavidin.^23^ Although this simple change in protein architecture gave CTB emergent fusogenic properties, mechanistic interpretation was hindered by structural heterogeneity, since Strep-(AB_5_)_*n*_ existed as mixtures with variable stoichiometry (*n* = 1–4) and orientation (e.g., cis- and trans-configurations of the divalent Strep-(AB_5_)_2_; Figure 1b).

**Figure 1:**
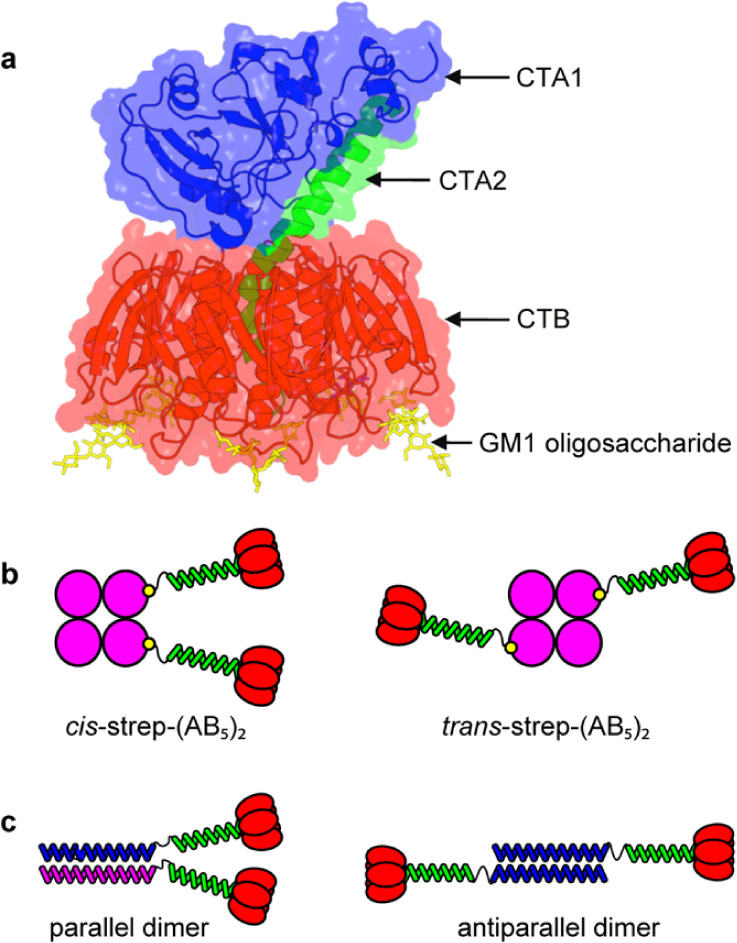
Membrane fusogens based on cholera toxin. (a) Overlay of ribbon and surface representations of cholera toxin holotoxin, reconstructed in PyMol by superimposing Protein Data Bank (PDB) entries 3CHB and 1XTC. The toxic CTA1 subunit, CTA2 linker peptide, and lectin CTB pentamer are shown in blue, green, and red, respectively. Bound ganglioside GM1 ligands are colored yellow. (b) Two configurations of Strep-(AB_5_)_2_ (*cis*- and *trans*-), which have membrane fusing activity. (c) Dimeric AB_5_ complexes linked by parallel and antiparallel coiled coils.

Here, we overcome these limitations by designing and synthesizing nontoxic AB_5_ complexes incorporating coiled-coil sequences to direct the controlled assembly of CTB dimers in either parallel or antiparallel orientations (Figure 1c). This approach allows us to dissect how coiled-coil orientation and CTA2 peptide length influence the fusogenic properties of CTB dimers.

## Results

### Design and Expression of CTB Dimer Assemblies

To generate CTB dimers, parallel or antiparallel coiled-coils were introduced as N-terminal extensions of the CTA2 peptide (Figure 2). Each coiled-coil construct was expressed as a fusion protein comprising a maltose-binding protein (MBP) affinity tag, the procoil sequence and CTA2 (Figure 2a–b). All MBP-procoil-CTA2 proteins were coexpressed with CTB and purified using sequential affinity chromatography: amylose agarose to capture all MBP-containing species, followed by Ni-NTA resin to select complexes containing CTB pentamers by binding to the His13 and His94 residues of CTB.^30^

**Figure 2:**
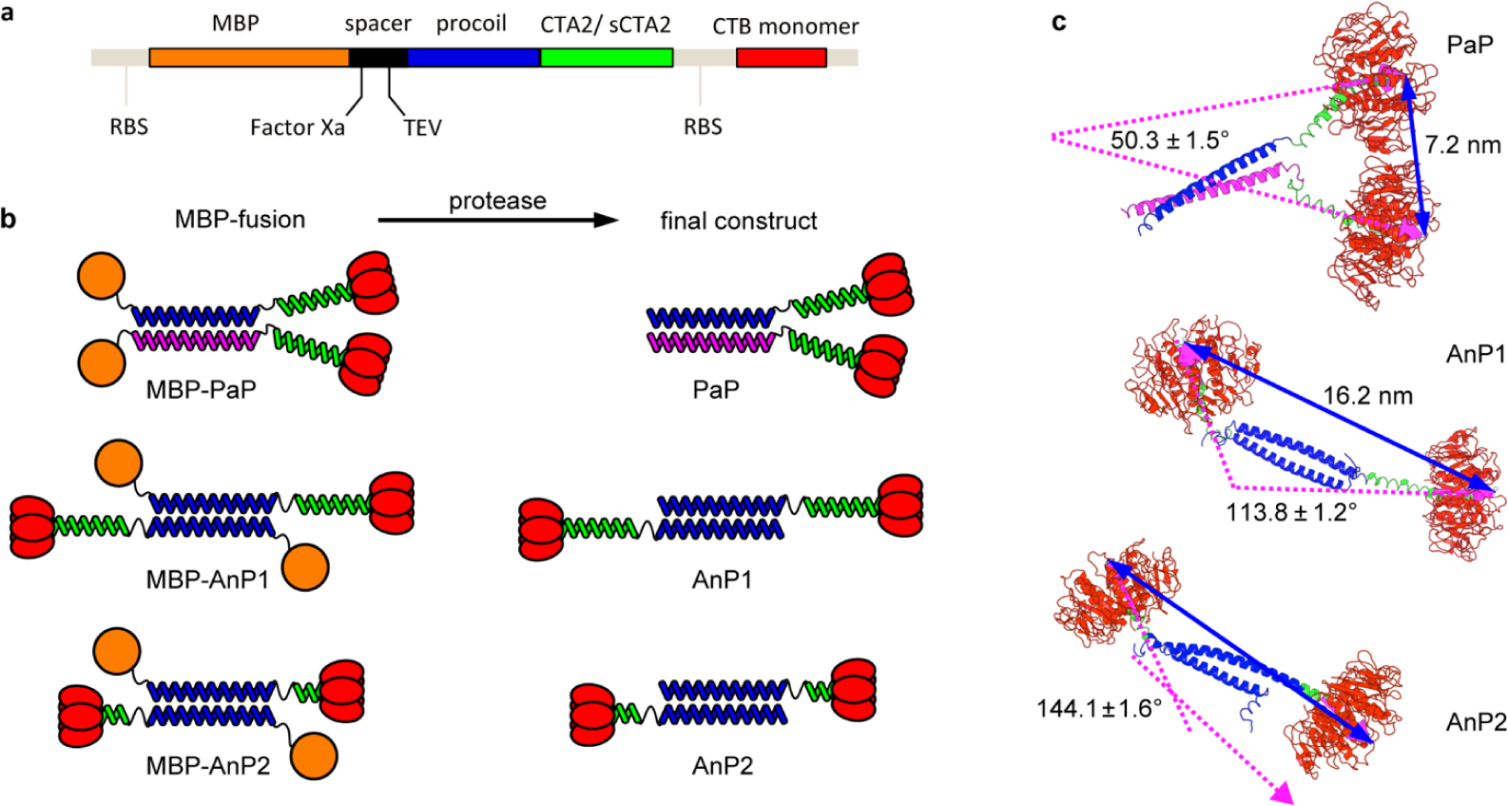
Coiled-coil constructs. (a) Schematic coding region of a bicistronic plasmid with two ribosome-binding sites (RBS) for coexpression of CTB and procoil-(s)­CTA2 fused to an N-terminal maltose-binding protein (MBP). (b) Three CTA2/CTB dimers designed in this study: PaP (heterodimeric parallel coiled-coil with full length CTA2), AnP1 (antiparallel coiled-coil with full length CTA2), and AnP2 (antiparallel coiled-coil with truncated CTA2: sCTA2). Each construct was initially expressed as an MBP fusion protein (MBP-PaP, MBP-AnP1, MBP-AnP2) and subsequently treated with TEV protease or Factor Xa protease to remove the MBP tag. (c) Representative models of AnP1, AnP2, and PaP (after MBP removal) showing median intersubunit distances and angles derived from Gaussian fits of simulation data. Angles were measured from vectors defined by His57 and Glu79 planes; distances were calculated between the centroids of His57 residues in each CTB pentamer.

After initial screening of several coiled-coil candidates, one heteromeric and two homomeric pairs were selected for detailed characterization based on their expression levels and ability to form stable dimers. The parallel-oriented CTB dimer (PaP = Parallel Protein), was designed using a heterodimeric parallel coiled-coil from Keating’s SYNZIP 1–2 pair (PDB code: 3HE5),^31^ which associates with sub-10 nM affinity.^31,32^ Two versions were made: one with an additional proline inserted between the SYNZIP and CTA2 sequences to separate the two helical domains, and one without the proline (PaP and PaP-noproline, respectively; Figures S1-1, S1-2). Two antiparallel CTB dimers (AnP1 and AnP2; “AnP” = Antiparallel Protein) were generated using homodimeric coiled-coils derived from sequences reported by Oakley^33^ and Marsh,^34^ respectively. To enhance expression, lysine residues on the noninteracting faces were replaced with glutamate, alanine or glutamine (Figure S1-1) to reduce ribosome stalling associated with lysine-rich sequences.^35^ Each procoil was fused to CTA2 to enable assembly with CTB (Figure S1-2). The full length CTA2 sequence was used for PaP to accommodate two bulky CTB pentamers in a parallel orientation. AnP1 also incorporated the full length CTA2, while AnP2 was linked to a shortened CTA2 peptide (sCTA2), producing a more compact antiparallel CTB dimer.

### Computational Modeling of CTB Dimers

The ISAMBARD software package was used to predict the topology and spatial arrangement of the coiled-coils used for the three CTB dimer constructs (Figure S2-1).^36^ The workflow began with a coarse screen to define the ISAMBARD parameter ranges (Figures S2-2, S2-3). The inbuilt genetic algorithm was then applied to each coiled-coil model in both parallel and antiparallel orientations, optimizing geometric parameters within this narrowed search space. The lowest-energy models were selected based on Bristol University Docking Engine (BUDE) energy scores and structural convergence (Figures S2-4, S2-5). This approach allowed BUDE energy scores to be compared directly, identifying the preferred orientation for each coiled-coil sequence. As expected, the AnP1 and AnP2 sequences strongly favored antiparallel orientations. For the PaP coiled-coil, the BUDE scores were similar for both orientations; however, the published crystal structure confirmed it adopts a parallel conformation.^31^

AlphaFold3 modeling was also in agreement with the expected coiled-coil orientations (Figure S2-6). The ISAMBARD-optimized coiled-coil models were then combined with the cholera toxin AB_5_ protein crystal structure (PDB: 1XTC). Molecular dynamics simulations (500–900 ns trajectories) were used to explore the conformational flexibility of the constructs. To evaluate the rigidity of the coiled-coil-CTA2 linkage, we measured the distances between the centroids of the His57 residues from each CTB pentamer, as well as the angles formed by vectors through their symmetry axes, throughout the simulations. These data were analyzed as histograms (Figure S3-1). The simulations indicated that in the PaP construct, the two CTB pentamers are held close together with an acute angle (median 50°) between their symmetry axis vectors. In contrast, both antiparallel constructs held the CTB subunits further apart; notably, the AnP2 design with the shortened linker favored a more parallel arrangement of the two CTB binding faces (Figure 2c). Together, these models reveal how coiled-coil orientation and linker length govern the spatial organization of CTB dimers, providing a structural framework for understanding their fusogenic activity.

### Biophysical Characterization of MBP-Linked Constructs

Biochemical analysis confirmed successful expression and assembly of the three MBP-linked CTB dimers (Figure 3). SDS–PAGE verified the integrity of the MBP–procoil–CTA2/CTB complexes, with CTB migrating as expected as a pentamer in unboiled samples and as monomers upon denaturation (Figure 3a). Oligomeric states were assessed by SEC and SEC–MALS (Figure 3b–c), which showed that both antiparallel constructs (MBP–AnP1 and MBP–AnP2) formed stable dimers. While the MBP–PaP.2-noproline component for the initial parallel coiled-coil design was clearly monomeric from the SEC-MALS data (Figure 3b), the complementary MBP–PaP.1-noproline component eluted more quickly (Figure 3c), indicating partial self-association which complicated interpretation of the coiled-coil assembly process. Insertion of a single proline residue between the SYNZIP domain and CTA2 to make MBP-PaP.1 and MBP-PaP.2 resolved this issue, providing monomeric proteins that assembled cleanly and reproducibly into parallel construct MBP–PaP as a heterodimer (Figure 3c). The proline-stabilized variant was therefore selected for all subsequent experiments.

**Figure 3:**
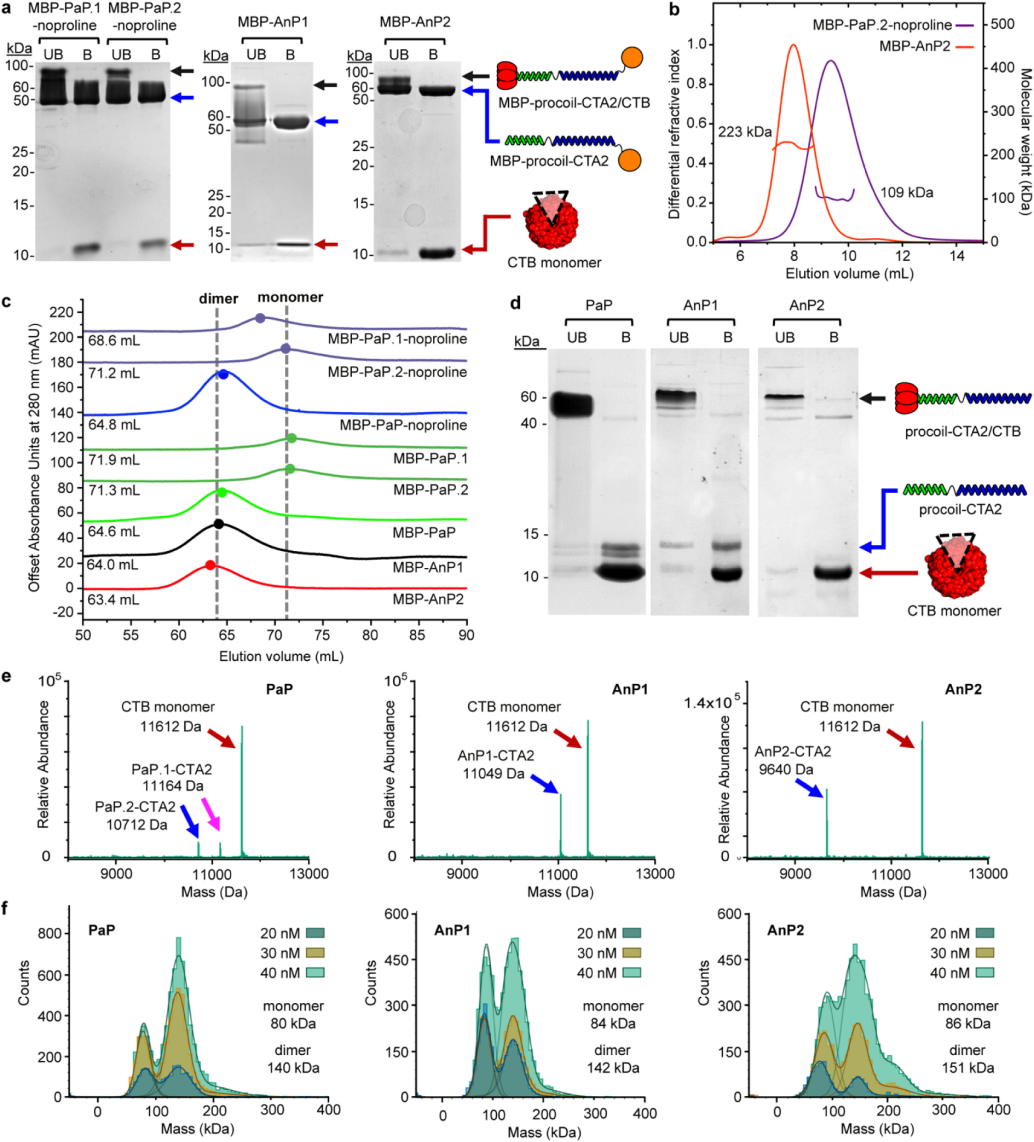
Characterization of CTA2/CTB dimers. (a) SDS-PAGE analysis of MBP-AnP1, MBP-AnP2, MBP-PaP.1-noproline, and MBP-PaP.2-noproline after sequential amylose and nickel affinity purification. Bands corresponding to AB_5_ complexes, A-subunits, and B-subunits are indicated (black, blue, and red arrows). “UB” = unboiled; “B” = boiled. (b) SEC–MALS analysis of representative monomeric and dimeric species. MBP-PaP.2-noproline eluted as a monomer (theoretical monomer mass = 112 822 Da; observed = 109 kDa ± 19%; *M*_w_/*M*_n_ = 1.002). MBP–AnP2 eluted as a dimer (theoretical monomer mass = 109 987 Da; observed = 223 kDa ± 6%; *M*_w_/*M*_n_ = 1.001). Chromatograms (refractive index) are overlaid with calculated molecular weights across each peak. (c) Size-exclusion chromatography (SEC) traces showing elution volumes for monomeric and dimeric species. MBP-PaP and MBP-PaP-noproline are shown as 1:1 mixtures of their component monomers (MBP-PaP.1/MBP-PaP.2 or MBP-PaP.1-noproline/MBP-PaP.2-noproline). (d) SDS-PAGE analysis of coiled-coil constructs after MBP removal by TEV or Factor Xa protease. For PaP, the PaP.1 and PaP.2 proteins were mixed 1:1 before analysis. “UB” = unboiled; “B” = boiled. In boiled samples, procoil-CTA2 and CTB monomers migrate separately; in unboiled samples, CTB remains pentameric and in complex with procoil-CTA2. For AnP2, the procoil-CTA2 band is faint in boiled samples (likely due to poor Coomassie staining), but unboiled samples migrate at ∼60 kDa, consistent with intact AB_5_ complexes, while CTB_5_ alone runs at ∼45 kDa. (e) Electrospray mass spectrometry confirming the presence of the expected subunits in each procoil-CTA2/CTB complex. (f) Mass photometry of AnP1, AnP2, and PaP showing concentration dependent mixtures of monomers and dimers at 20–40 nM, indicating low nanomolar dissociation constants.

### Removal of MBP Tags and Multimerization Analysis

The MBP domain was removed using site-specific proteolysis. Tobacco Etch Virus (TEV) protease efficiently cleaved MBP from the PaP and AnP1 constructs, but was ineffective for the AnP2 construct (Figure S4-1), presumably due to steric occlusion of the cleavage site by either the coiled-coil or its shortened CTA2 linker. This issue was resolved by switching to Factor Xa protease, which enabled complete tag removal from AnP2.

The resulting proteins were purified by amylose chromatography to remove the MBP tag, followed by nickel chromatography to separate the AB_5_ construct from the protease. SDS-PAGE (Figure 3d) and mass spectrometry (Figure 3e) confirmed the presence of the expected components for each construct. Mass photometry, at lower concentrations (20–40 nM dimer), revealed that all three coiled-coil dimers exist in a dynamic equilibrium between monomeric and dimeric species, with the proportion of dimeric species increasing with concentration (Figure 3f). This experiment showed the three coiled-coils had similar dissociation constants (*K*_d_) in the low nanomolar range, and that despite differences in sequence, orientation and linker length, each construct will form a stable CTB dimer at the concentrations used in subsequent experiments.

### Lipid Mixing Assay Reveals Different Fusion Behaviors for CTB Dimers

The fusogenic activities of AnP1, AnP2, and PaP were compared using a lipid mixing assay based on Förster resonance energy transfer (FRET) between lipid-linked NBD and rhodamine dyes (Figure S5-1).^37^ Labeled large unilamellar vesicles (FRET-LUVs; DOPC:DOPE:cholesterol:GM1:PE-NBD:PE-Rho = 47:24:24:5:0.25:0.25) were mixed with unlabeled vesicles of identical composition (blank-LUV; DOPC:DOPE:cholesterol:GM1 = 48:24:24:5), both 800 nm in diameter at a final lipid concentration of 133 μM. Lipid exchange between FRET-LUVs and blank-LUVs increases the average distance between dyes (Figure 4a), leading to dequenching of NBD fluorescence. The extent of dequenching, expressed as the 520/585 nm fluorescence ratio, was used to calculate the percentage and rate of lipid mixing (Figures 4b and S5-2). Control reactions showed minimal lipid mixing in the absence of CTB dimers or with CTA2/CTB lacking procoils (Figure 4b). In contrast, PaP, AnP1, and AnP2 all promoted concentration-dependent lipid mixing, with reproducible differences between constructs. PaP and AnP1 showed maximal activity near equimolar concentrations of GM1 and CTB binding sites (∼1 μM), followed by reduced activity at higher concentrations which is consistent with saturation of binding sites that inhibits vesicle cross-linking.^38^ In contrast, AnP2 exhibited a monotonic increase in lipid mixing efficiency until reaching a plateau (Figure 4b). These results demonstrate that CTB oligomerization promotes lipid mixing, but linker length may be more significant than orientation for modulating lectin-driven fusion behavior.

**Figure 4:**
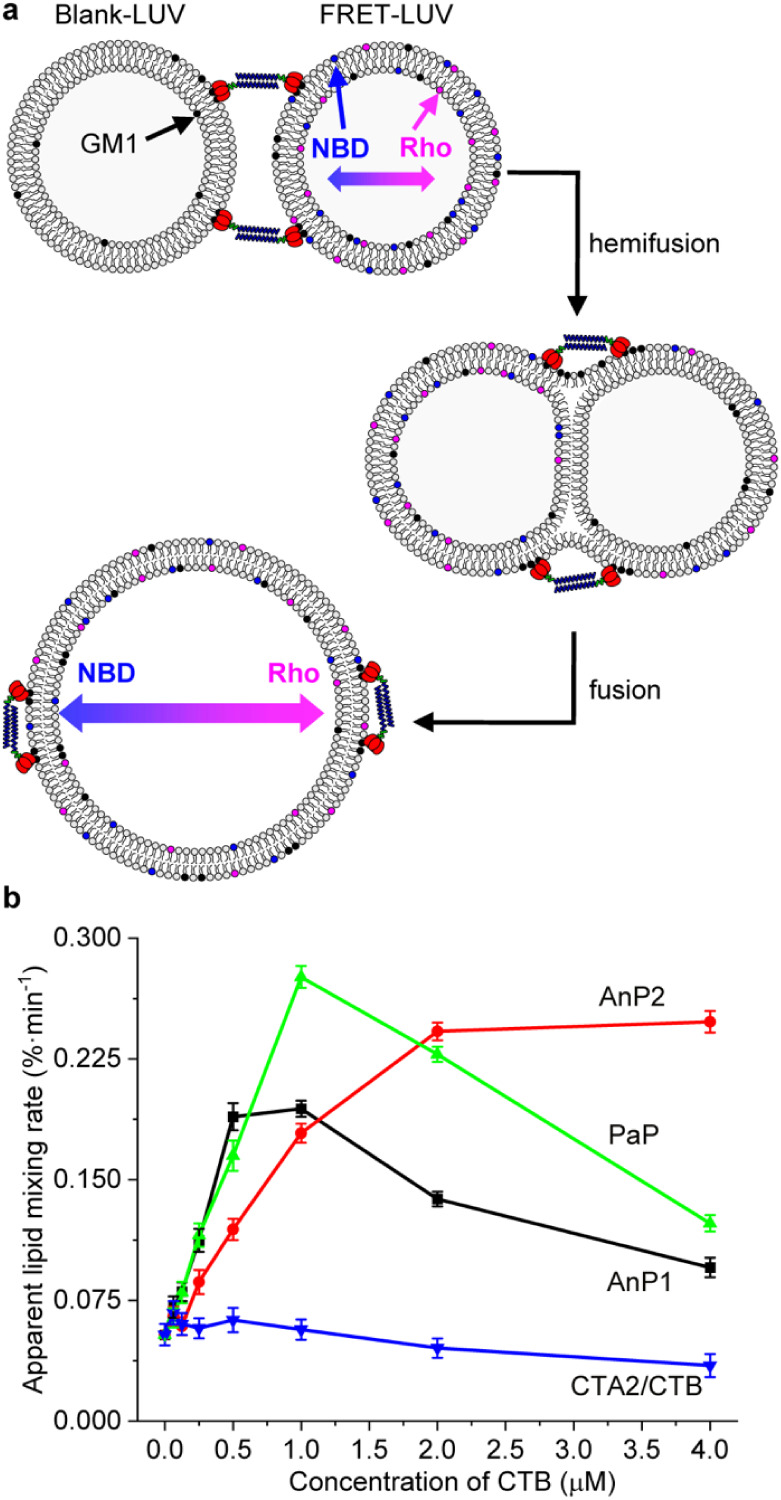
Lipid mixing assays of CTB dimer complexes. (a) Schematic representation of the lipid mixing assay. Blank LUVs contain GM1 (black dots), while FRET-LUVs contain GM1 together with the FRET donor NBD (7-nitro-2,1,3-benzoxadiazol-4-yl; blue dots) and acceptor sulforhodamine B (Rho; pink dots). Lipid exchange between FRET-LUVs and Blank-LUVs increases the distance between donor and acceptor dyes, leading to reduced FRET signal. (b) Apparent lipid mixing rates, presented as percentage of lipid mixing per minute, plotted against CTB pentamer concentration. (*n* = 3, mean ± SD).

### Observing Giant Unilamellar Vesicle (GUV) Fusion

While lipid mixing assays provided population-level evidence for lipid exchange, confocal microscopy of GUVs was used to directly distinguish between lipid exchange, full fusion, and rupture events. Two GUV populations containing 5 mol % GM1 were prepared: one labeled with 0.3 mol % DOPE-Atto647N (Atto647N-GUVs) and one unlabeled (Blank-GUVs). Proteins were added at a concentration of 400 nM CTB pentamer (200 nM CTB dimers), conjugated to Alexa Fluor 488 for visualization. A total of 410, 397, and 503 GUVs were counted separately for AnP1, AnP2, and PaP, respectively, across several experiments to quantify the lipid exchange, fusion and rupture events per 100 vesicles (Figure 5a). Lipid exchange was recorded when the DOPE-Atto647N transferred from labeled to unlabeled GUVs, fusion when two vesicles merged into one, and rupture when vesicles burst. No lipid exchange or rupture was observed in the absence of added protein (Figure S6-1).^23^ AnP2 treatment produced membrane cross-linking and fusion (Figure 5a,c; Video SV1), with 11% of GUVs showing lipid exchange and fusion. AnP1 and PaP induced comparable lipid exchange frequencies (15% and 19%, respectively) but markedly fewer fusion events (3% and 1%, respectively; Videos SV2 and SV3). Representative examples for each construct include gradual interface elongation prior to fusion with AnP1 (p1; Figure 5b), two sequential fusion events triggered by AnP2 (p1 and p2; Figure 5c), and progressive lipid dye redistribution between vesicle populations (p1 and p2; Figure 5d). Together, these results reveal that although all CTB dimers can drive lipid exchange, the AnP2 architecture provides the most efficient GUV fusion, demonstrating that linker truncation can bias lectin assemblies toward productive fusion rather than mere lipid transfer.

**Figure 5:**
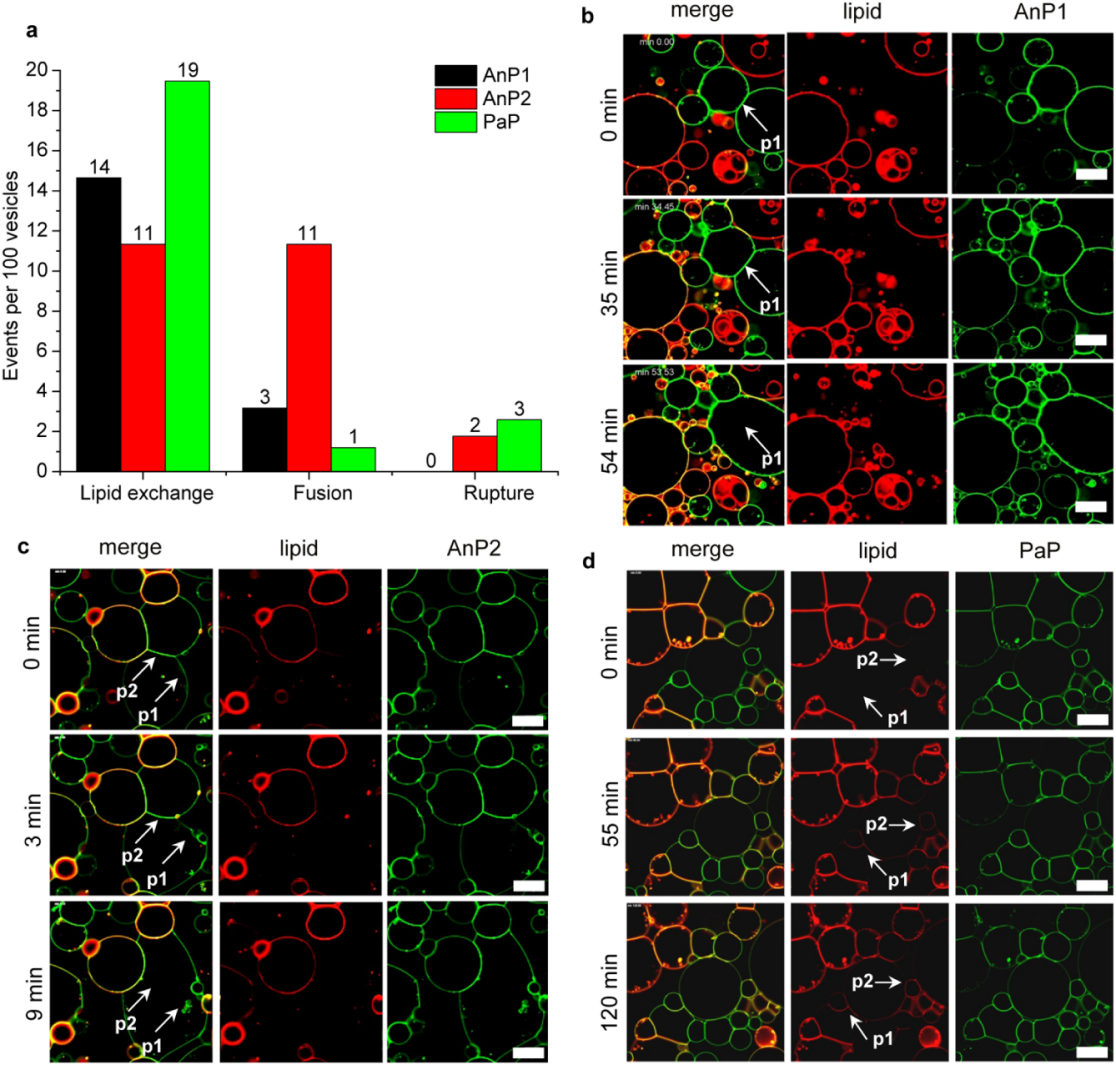
GUV behaviors induced by AnP1, AnP2, and PaP. (a) Quantification of lipid exchange, fusion, and rupture events per 100 GUVs. Data were extracted from 410, 397, and 503 vesicles for AnP1, AnP2, and PaP, respectively, across multiple experiments. Fusion events are included in both the “Fusion” and “Lipid exchange” categories, as not all lipid exchange necessarily results in fusion. (b–d) Time-lapse confocal microscopy images of two GUV populations containing 5 mol % GM1: Blank-GUVs without dye and Atto647N-GUVs with 0.3 mol % DOPE–Atto647N (red). Vesicles were incubated with 200 nM AB5 dimers labeled with AF488 (green). All three CTB dimers induced extensive membrane cross-linking, evident from elongated interfaces and distorted vesicle shapes. Full fusion was observed for AnP1 and AnP2 (white arrows), while PaP primarily caused cross-linking and lipid dye transfer from labeled to unlabeled GUVs (white arrows). Full time series are shown in Supporting Information Videos 1–3. Scale bars, 20 μm.

### Analysis of Membrane Interactions by Quartz Crystal Microbalance

We used quartz crystal microbalance with dissipation monitoring (QCM-D) to probe the binding modes of CTB-dimers to GM1-containing membranes (Figure 6a). Small unilamellar vesicles (SUVs; typically 30 nm) comprising 5 mol % GM1 and 95 mol % DOPC were prepared and used to construct supported lipid bilayers (SLBs) on silica sensors. CTB dimer complexes were flowed over the SLBs to allow binding to GM1, before adding more GM1-SUVs to test whether immobilized CTBs could interact with an additional membrane. Representative data from three replicates are shown in Figure 6b, with full data sets in Figure S7-1.

**Figure 6:**
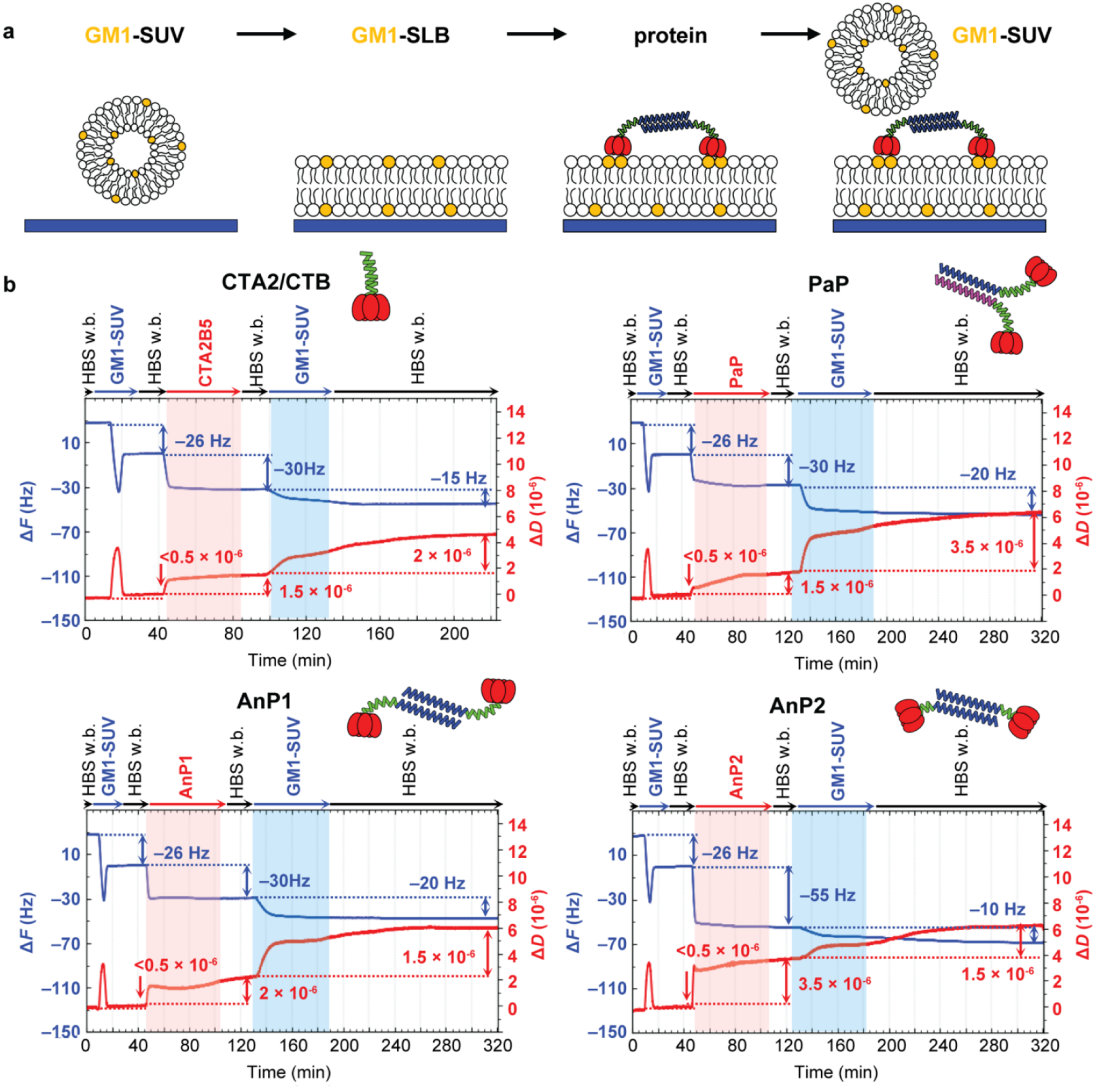
QCM-D analysis of CTB dimer binding to GM1-containing membranes. (a) Schematic diagram of the QCM-D experiment: formation of a GM1 supported lipid bilayer (SLB) from GM1-SUVs on the sensor surface; binding of CTB proteins to the GM1-SLB; addition of a second layer of GM1-SUVs to probe outward-facing binding sites. (b) Representative QCM-D sensorgrams showing the interactions of CTA2/CTB, PaP, AnP1, and AnP2 with GM1-SLBs, followed by the addition of GM1-SUVs. Frequency shifts (Δ*F*, blue) reflect mass loading (including bound solvent), while dissipation shifts (Δ*D*, red; overtone *i* = 5) report on layer softness. Shaded regions highlight the protein binding phase (light red) and the subsequent GM1-SUV addition (light blue). Arrows mark sample incubation steps or washing with HEPES-buffered saline working buffer (HBS w.b.). Introduction of 50 μg/mL GM1-SUV (5 mol % GM1 + 95 mol % DOPC) at flow rate 20 μL/min, resulted in a Δ*F* = −26 ± 1 Hz and Δ*D* < 0.5 × 10^6^, indicating successful formation of a high-quality GM1-SLB. CTB dimers were introduced at 200 nM (equivalent to 400 nM CTB pentamer), followed by a buffer wash and final addition of GM1-SUVs. PaP and AnP1 behaved similarly to CTA2/CTB, while AnP2 showed distinct binding and viscoelastic signatures, revealing that linker architecture controls the membrane orientation and flexibility of CTB dimers.

#### SLB Formation and CTA2/CTB Binding

Initial exposure of GM1-SUVs to the sensor surface, produced the characteristic Δ*F* and Δ*D* spikes corresponding to vesicle adsorption, rupture and spreading into an SLB (Figure 6b).^39^ Initial binding of intact SUVs entails a large negative frequency shift owing to trapped solvent, and an increase in dissipation owing to their soft appearance, upon shear oscillation of the QCM-D sensor surface. Following SUV rupture and SLB formation, the trapped solvent is released and the surface-coupled lipid bilayer has virtually no dissipation. The frequency shift of −26 Hz for the SLB corresponds to a layer thickness of 5.0 nm (see Methods for details), consistent with expectations for a lipid membrane containing a small fraction of rather bulky GM1 headgroups. Addition of CTA2/CTB led to rapid and stable binding (Δ*F* = −30 Hz), corresponding to a protein layer thickness of ∼5 nm. This value is slightly lower than the expected ∼6 nm predicted from the crystal structure (PDB: 1XTC), and is consistent with the flexibility of the CTA2 peptide observed during MD simulations (Figure S3-1). Introduction of GM1-SUVs caused further changes in frequency and dissipation (Δ*F* ≈ −15 Hz, Δ*D* ≈ 2 × 10^–6^; Figure 6b, from 98 min). As no binding was observed for DOPC-only SUVs (Figure S7-2), this behavior indicated that a subset of CTB sites remained accessible to a second membrane. However, these values were far smaller than those obtained with a control construct designed to orient CTB binding sites away from the membrane (CTB-H_6_,^40^ with C-terminal his-tags bound via Ni-NTA; Δ*F* = −90 Hz, Δ*D* = 8 × 10^–6^; Figure S7-3), demonstrating that most CTA2/CTB binding sites were engaged with the SLB surface.

#### Comparing CTB Dimers

PaP and AnP1 exhibited frequency shifts similar to CTA2/CTB alone (Δ*F* ≈ −30 Hz), suggesting both CTB units bound simultaneously to the SLB. In contrast, AnP2 produced a much larger frequency shift (Δ*F* = −55 Hz) and higher dissipation (Δ*D* = 3.5 × 10^–6^), consistent with greater structural flexibility. This result was surprising as AnP2 has a shorter A2 linker peptide than AnP1, but forms a thicker layer on the GM1-SLB. One explanation is that the shortened CTA2 linker prevents both CTB units from binding to the SLB, leading to an “upright” orientation with one CTB unit bound and the other pointing away from the surface. If this were the case, then AnP2 should have the greatest capacity for binding to additional GM1-SUVs in the final phase of the experiment. However, when GM1-SUVs were introduced to the system, AnP2 bound less (Δ*F* = −10 Hz and Δ*D* = 1.5 × 10^–6^) than CTA2/CTB (Δ*F* = −15 Hz and Δ*D* = 2 × 10^–6^), PaP and AnP1 (Δ*F* ≈ −20 Hz and Δ*D* ≈ 3 to 3.5 × 10^–6^), and much less than those for CTB-H_6_ on the Ni-NTA surface (Figure S7-3). These observations argue against the “upright” configuration being the dominant state and support a model in which both CTB pentamers principally adopt surface-bound orientations. Complementary AFM imaging of all three dimers on nickel-treated mica surfaces (Figure S8-1) showed pairs of CTB pentamers lying flat with mean interpentamer distances of 6.25–6.69 nm. This result shows that even weak interactions between the protein and surface favor surface-bound orientations of CTB pentamers.

An alternative explanation is that in AnP2, the shorter linker would be too strained for both CTB units to bind the GM1-SLB at the same time, so binding can only occur if the coiled-coil dissociates. A “dissociated complex” scenario could result in an unstructured procoil with an expanded hydration shell which would be consistent with the greater frequency and dissipation changes for AnP2 (Δ*F* = −55 Hz; Δ*D* = 3.5 × 10^–6^), and it having no greater ability to bind GM1-SUVs than the CTA2/CTB complex.

The QCM-D experiments show that PaP and AnP1 exhibit similar binding to GM1-containing membranes, whereas AnP2—with its shorter linker—potentially adopts a distinct surface configuration. These differences in membrane interaction can be expected to influence their fusogenic potential.

### Jurkat Cell Interactions

We next examined how CTB complexes interact with mammalian cells (Figure 7). As previous experiments had consistently indicated that activity depended more on CTA2 linker length than on coiled-coil orientation, we focused on AnP1, AnP2 and the CTA2/CTB control. Each protein complex was labeled with AF488 before incubation with Jurkat cells at concentrations between 0.25 nM and 40 nM (based on CTB pentamer concentration). Flow cytometry revealed AnP1 and AnP2 reached saturation at lower concentrations than CTA2/CTB (EC_50_ = 1.55 ± 0.15 nM and 1.29 ± 0.16 nM vs 3.45 ± 0.29 nM, respectively; Figure 7a–b, Figure S9-1), and with higher Hill slopes (*n* = 2.0 vs *n* = 1.5), consistent with increased avidity from their dimeric structures.

**Figure 7:**
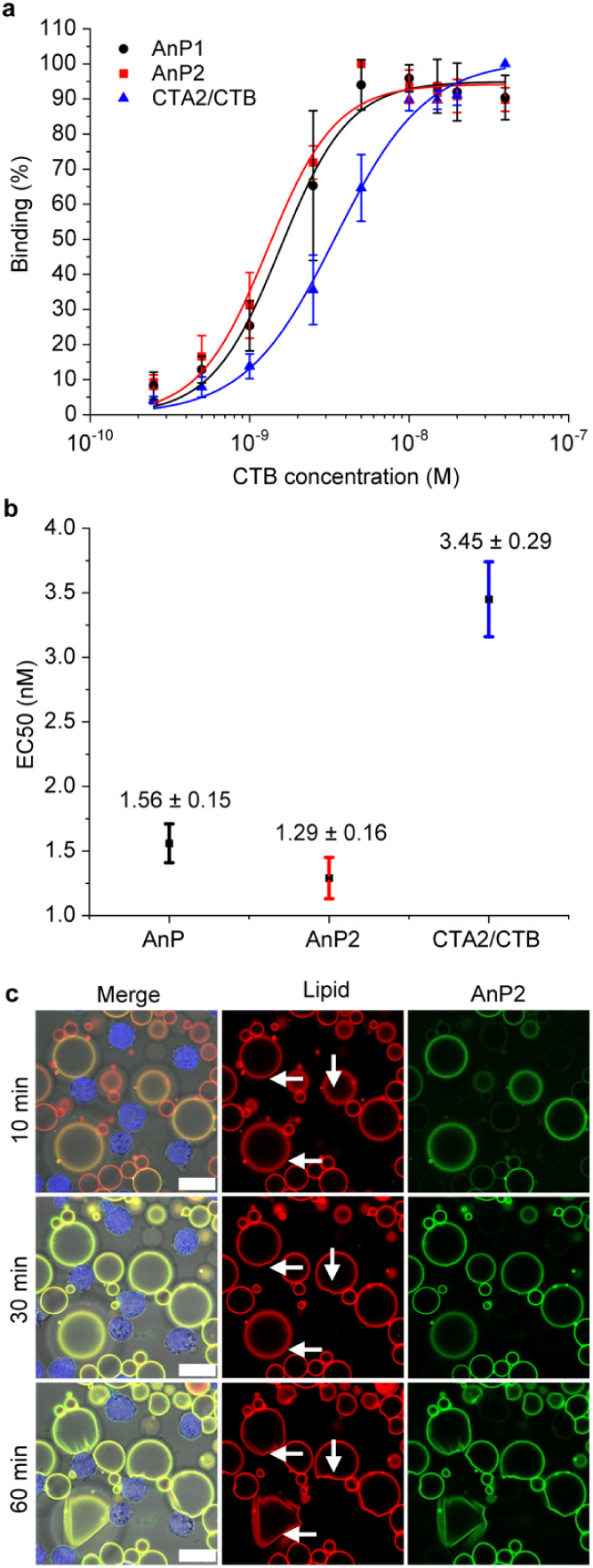
Binding of CTA2/CTB dimers to Jurkat T cells and induction of cell–GUV cross-linking. (a) Normalized flow cytometry fluorescence intensities (*n* = 4, mean ± SD) versus protein concentration, fitted to the Hill equation. (b) Half-maximal effective concentrations (EC_50_) of CTA2/CTB, AnP1, and AnP2 binding to Jurkat cells. (c) Confocal microscopy images of Jurkat cells (blue, CellTrace Violet) incubated with GM1-GUVs (red, DOPE–Atto647N) and AnP2 (green, AF488). Addition of AnP2 (200 nM dimer; 400 nM CTB pentamer) induced cross-linking between GUVs and cells, and deformation of GUV membranes (white arrows). Imaging was performed at 37 °C for 60 min starting at protein addition. Full time series are available in Videos SV6–SV8. Scale bar, 20 μm.

We then asked whether dimeric CTB complexes could mediate interactions between Jurkat cells and GM1-containing GUVs. In the absence of protein, or in the presence of CTA2/CTB or AnP1 (400 nM CTB pentamer), GUVs remained spherical and did not cross-link with cells (Figure 7c, Figures S10-1, S10-2 and Videos SV4, SV5, SV6, SV7). In contrast, AnP2 at the same concentration induced visible cross-linking after 1 h, leading to deformation of GUVs in contact with the Jurkat cells (Figure 7c and Video SV8). The fluorescent signal localized mainly to GUVs, presumably reflecting the higher concentration of GM1 on their surface; however, faint AnP2 binding to cell membranes out with cross-linked interfaces was detected after 1 h (Figure 7c). No transfer of DOPE-Atto647N lipids from GUVs to cells was observed. Nonetheless, the results are qualitatively in line with the other experiments that AnP2 behaves differently from AnP1 and CTA2/CTB in its interactions with GUVs.

## Discussion

In our earlier work, we showed that multimeric complexes of CTB could mediate fusion of GM1-containing GUVs,^23^ with fusogenicity arising from CTB’s intrinsic membrane-bending activity combined with cross-linking of GM1-decorated membranes. In the current study we sought to dissect the factors that affect fusogenic behavior by assembling defined CTB dimers using coiled-coils of different orientations. Although we expected orientation (parallel vs antiparallel) to play the dominant role, our results consistently revealed that linker length and flexibility were more critical determinants. Specifically, AnP1 and AnP2 (both antiparallel dimers) exhibited more divergent behaviors from each other than AnP1 from the parallel PaP construct. Thus, the length of the CTA2 linker connecting CTB units, rather than coiled-coil orientation, emerged as the principal factor controlling fusogenic outcomes. This finding highlights that linker engineering may be a general design principle for programming lectin-mediated membrane fusion.

All three CTB dimers supported lipid exchange in FRET-based lipid mixing assays. For AnP1 and PaP, lipid mixing efficiency increased with protein concentration up to an equimolar ratio of GM1 to CTB binding sites (∼1 μM CTB pentamer, 5 μM binding sites; Figure 4b), after which efficiency declined. In contrast, AnP2 displayed a monotonic increase in lipid mixing, reaching a plateau at the highest concentrations tested (up to 20 μM CTB monomer, i.e., 4 μM pentamer). As the lipid mixing assay cannot distinguish between lipid exchange, full fusion and membrane rupture events, we also studied the proteins using GUVs and confocal microscopy (Figure 5). All three dimers mediated cross-linking, lipid transfer and fusion events, but AnP2 again stood out by producing markedly more fusion events per 100 GUVs analyzed.

It is important to note that the assays differed substantially in stoichiometry. The lipid mixing experiments used a bulk lipid concentration of 133 μM, corresponding to 6.65 μM GM1, with CTB monomer concentrations from 0–20 μM (ratios up to ∼3:1 CTB:GM1). In contrast, the GUV experiments employed ∼2.5 nM effective lipid concentration and 2 μM CTB monomer (400 nM pentamer), giving an estimated CTB:GM1 ratio of ∼800. Direct quantitative comparison between the two systems is therefore not appropriate. Nevertheless, across both assays AnP2 consistently promoted more disruptive events, with combined fusion/rupture frequencies of 4:3:13 per 100 GUVs for PaP, AnP1, and AnP2, respectively.

To understand the different behaviors of the three constructs, we considered how each protein interacts with the membranes. Flow cytometry showed that AnP1 and AnP2 bound Jurkat cells with lower EC_50_ values than CTA2/CTB. If the dimers adopted an “upright” configuration, with only one CTB pentamer contacting the membrane (Figure 8a), their apparent affinity would be expected to decrease, giving EC_50_ values approximately twice that of CTA2/CTB. Instead, the opposite trend was observed: both AnP1 and AnP2 bound more strongly than CTA2/CTB. This indicates that the dimers interact with membranes in a “reclining” orientation (Figure 8b) where both CTB pentamers engage GM1 simultaneously, and that coiled-coil-mediated dimerization enhances avidity through increased valency.

**Figure 8:**
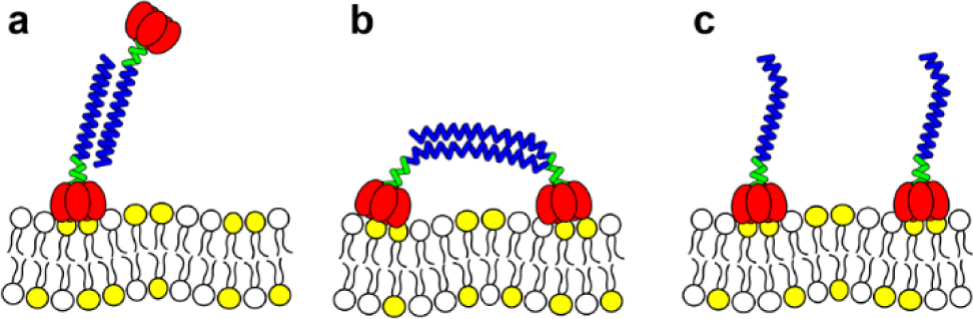
Models of CTB dimer–membrane interactions. Three potential binding states are illustrated: (a) Upright—one CTB pentamer adheres to the membrane while the second projects into solution. (b) Reclining—both CTB pentamers simultaneously engage GM1 while remaining linked by the coiled-coil. (c) Dissociated—the coiled-coil separates, allowing both CTBs from a dimer to bind independently to the membrane.

QCM-D experiments provided complementary evidence for the binding modes of CTB dimers. When each construct was bound to a GM1-supported lipid bilayer (SLB) and challenged with GM1-SUVs, only limited additional binding was detected (Δ*F* = −15 to −20 Hz). This was much lower than for the CTB-H6 control on a Ni–NTA SLB (Δ*F* = −90 Hz; Figure S7-3), which orients its binding sites outward because it has five hexa-histidine tags arranged on its C-terminal face that is distal to the GM1 binding sites. Thus, the majority of CTB units in the coiled-coil dimers were engaged with GM1 on the SLB surface.

Interestingly, even CTA2/CTB alone showed some capacity to bind GM1-SUVs (Figures 6b and S7-1). This result was surprising as AFM studies have indicated that CTB sits flat on a GM1-SLB,^41^ but it is possible that once most GM1 ligands have been bound by CTB in a flat orientation, any remaining GM1 might capture additional CTB pentamers in a “side-on” orientation. We do not assume that a “side-on” binding orientation necessarily extends to GUVs, as these are less rigid than SLBs and CTA2/CTB showed no activity in lipid mixing assays (Figure 4). Nevertheless, because all three dimers cross-linked GUVs in confocal microscopy experiments, some fraction of CTB units must orient outward to enable vesicle–vesicle bridging. For AnP1 and AnP2 this could involve a minority of dimers in an “upright” orientation, while for PaP it may reflect a small proportion of molecules adopting an antiparallel arrangement. The ISAMBARD/BUDE calculations indicated that such an arrangement is possible for PaP, even though the parallel orientation is preferred (Figure S2-5).

QCM-D experiments also revealed clear differences in how the constructs behave when bound to the membranes. PaP and AnP1 caused frequency shifts similar to those for CTA2/CTB (−30 Hz). In contrast AnP2 displayed a much greater frequency shift of −55 Hz, and higher dissipation, corresponding to a thicker and softer layer. This was surprising, as AnP2 has the shortest linker between the pentamer and coiled-coil. It was also the least able to capture additional GM1-SUVs, suggesting that both CTB pentamers were bound to the SLB. We therefore propose that the coiled-coil in AnP2 destabilizes and unravels upon membrane engagement (Figure 8c), and this phenomenon gives rise to its distinctive QCM-D signature and its enhanced fusogenic activity relative to PaP and AnP1.

While QCM-D provides strong evidence for the formation of a thicker and more dissipative layer, it cannot by itself unambiguously distinguish between coiled-coil and lipid bilayer reorganization. Both processes can contribute to larger negative frequency shifts and increased dissipation. However, the rapid equilibration upon AP2 addition and absence of progressive viscoelastic changes typically associated with SLB restructuring,^42^ make extensive lipid reorganization less likely in this case, and support our interpretation that AnP2 undergoes coiled-coil destabilization upon membrane binding.

The concentration dependence of lipid mixing can be explained by considering how the dimers engage GM1. For PaP and AnP1, both CTB units initially bind to GM1 on the same vesicle (Figure 9). Cross-linking to a second vesicle occurs more slowly, causing the lipid mixing rate to increase with protein concentration up to an equimolar ratio of CTB binding sites to GM1. Beyond this point, the ligand becomes saturated, leaving too few free sites to mediate productive cross-linking,^38^ and lipid mixing efficiency declines.

**Figure 9:**
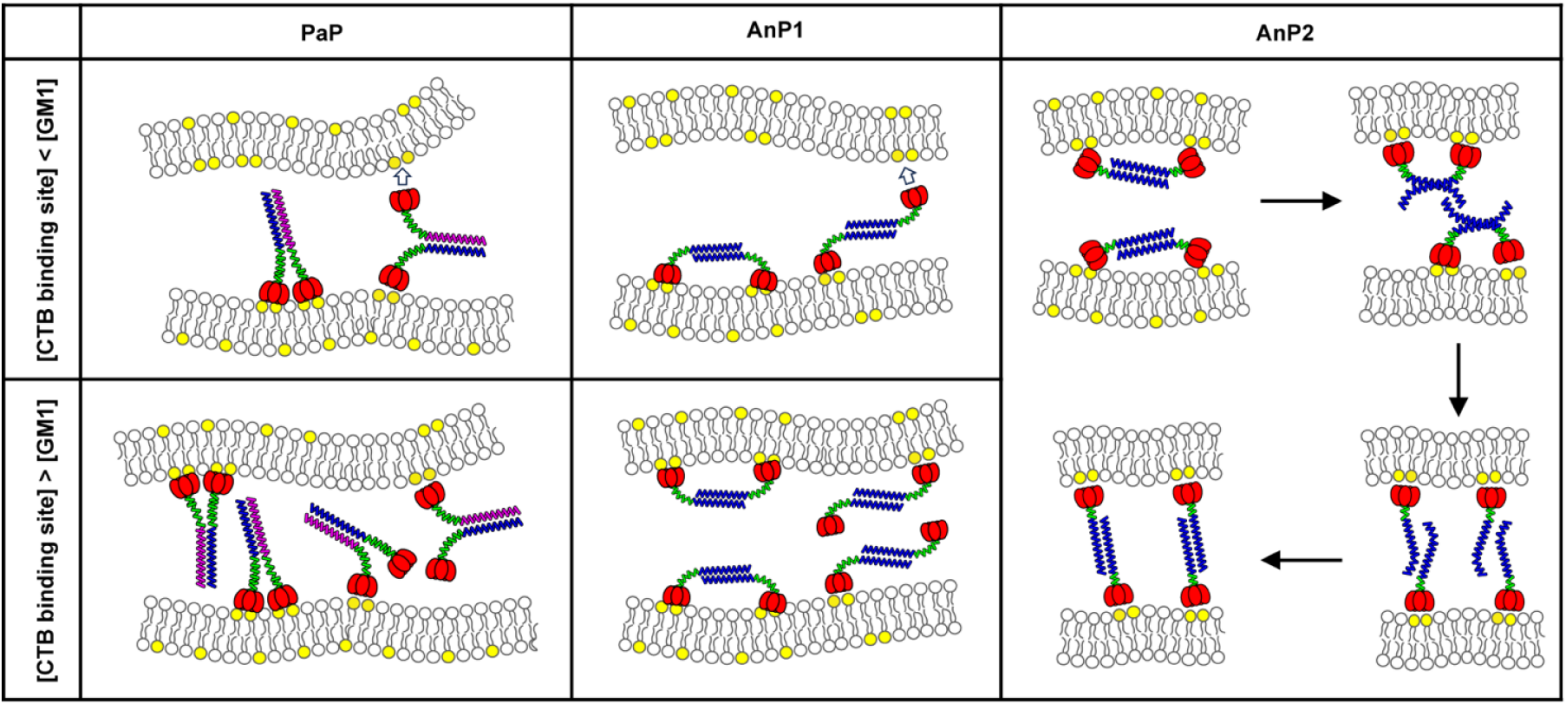
Proposed mechanisms of membrane fusion mediated by PaP, AnP1, and AnP2. For PaP and AnP1, at low CTB:GM1 ratios, the full-length CTA2 linker provides flexibility that allows free CTB binding sites to reach neighboring membranes, facilitating cross-linking and fusion. At higher CTB concentrations, GM1 saturation blocks bridging and reduces fusion efficiency. In contrast, AnP2, with its shorter CTA2 linker, is prone to coiled-coil unwinding upon membrane binding. The resulting dissociated procoils can reassociate with procoils on adjacent membranes, enabling “reassembled” cross-links that continue to promote fusion even under GM1-saturated conditions.

In contrast, AnP2, with its shorter CTA2 linker, likely experiences greater strain when both CTBs bind simultaneously to the same membrane. QCM-D results are consistent with the idea that the coiled-coil in AnP2 destabilizes upon binding, yielding flexible, unraveled procoils (Figure 9). These exposed helices could reassociate with procoils from neighboring vesicles, driving cross-linking and fusion independently of GM1 availability. This mechanism provides an explanation for why AnP2 maintained lipid mixing activity at higher concentrations, whereas PaP and AnP1 did not.

The same logic extends to cell–GUV interactions. Although AnP1 and AnP2 bound Jurkat cells with similar affinity, only AnP2 induced GUV deformation and cross-linking. Jurkat cells express relatively low levels of GM1, leading to a high CTB:GM1 ratio, and their glycocalyx may further restrict protein binding. Under these conditions, AnP1 would saturate GM1 sites without enabling bridging. By contrast, AnP2 could still promote cross-linking through coiled-coil dissociation and reassembly with procoils on GUVs, thereby overcoming GM1 limitation.

These results highlight a key distinction: PaP and AnP1 rely on GM1 ligand availability for bridging, which is sensitive to stoichiometry, while AnP2 exploits coiled-coil dissociation to drive membrane cross-linking even when free GM1 is scarce.

Beyond these mechanistic insights, the behavior of AnP2 highlights a broader design concept. Membrane-induced coiled-coil dissociation–reassociation also presents the possibility of engineering homodimeric lectins that assemble into functional heterotypic bridges when two membranes carrying distinct glycan receptors are brought together. This mechanism could be adapted to create glycan-dependent pairing between different vesicle populations or cell types, extending lectin-based fusogens from simple cross-linkers to programmable, multi-input recognition elements.

More broadly, GM1-targeting fusogens offer functional advantages that complement these programmable architectures. CTB binds GM1 with high affinity on diverse cell types, including mucosal epithelial cells and neurons, where CTB-mediated targeting is already well established.^43–45^ By engineering CTB dimers whose fusogenic behavior can be tuned through coiled-coil architecture, this work extends the utility of GM1-binding platforms from passive targeting toward *programmable interfacial remodeling*. Compared with approaches based on pH-responsive lipids, generic fusogenic peptides, or electrostatic interactions, GM1-specific lectin fusogens offer a unique combination of molecular specificity for natural cell surface ligands, genetic encodability, and structural modularity. These properties position CTB-derived constructs as promising tools not only for targeted delivery, but also for controlled membrane engineering, vesicle–cell hybrid assembly, and synthetic biological systems that require precise, receptor-defined fusion events.

## Conclusion

In this study, we engineered three dimeric CTB constructs using parallel (PaP) or antiparallel (AnP1, AnP2) coiled-coils with different linker lengths, and characterized them by molecular modeling, biophysical analysis, and functional assays. All three were able to cross-link GM1-containing vesicles and promote membrane fusion. PaP and AnP1 behaved similarly across lipid mixing, GUV fusion, and QCM-D assays, whereas AnP2 showed distinct behavior, maintaining fusogenic activity at higher CTB:GM1 ratios, promoting more efficient GUV fusion, and uniquely cross-linking vesicles to Jurkat cells. QCM-D analysis suggested that AnP2’s shorter linker destabilizes the coiled-coil upon membrane binding, enabling procoil dissociation and reassembly across membrane interfaces. Together, these results reveal that CTA2 linker length, rather than coiled-coil orientation, is the key determinant of fusogenic activity for CTB dimers. Robust membrane bridging can be achieved either through protein–carbohydrate interactions (PaP, AnP1) or through coiled-coil reorganization (AnP2). By demonstrating how subtle changes in lectin design alter fusogenic outcomes, this work establishes general principles for engineering lectin-based fusogens and takes a step toward their application in next-generation delivery and bioengineering strategies. The dissociation–reassociation mechanism in particular offers a route to create lectins that form heterotypic, glycan-dependent bridges between distinct vesicle or cell populations. In combination with CTB’s established ability to target GM1-rich neurons and mucosal epithelial cells, these design features enable selective deployment of fusogens to defined biological interfaces. Together, our findings establish a modular framework for constructing CTB-based fusogens with programmable specificity, expanding their potential across targeted delivery, membrane engineering, and the assembly of hybrid vesicle–cell systems.

## Experimental Section

### Protein Expression and Purification

MBP–procoil–CTA2/CTB constructs were generated by modifying the previously described pTRBAB5-G1S plasmid, which encodes MBP–CTA2 and CTB.^23^ DNA fragments encoding the procoil–CTA2 variants were synthesized (Twist Bioscience) with BamHI and XhoI restriction sites and ligated into the plasmid to replace the native CTA2 sequence. Recombinant plasmids were transformed into *E. coli* C41­(DE3) cells, which were grown in LB medium at 37 °C to OD_600_ = 0.6–0.8, before induction with IPTG (0.5 mM) and expression at 30 °C for 20 h. Proteins secreted into the extracellular medium were collected by centrifugation (10,000 × *g*, 10 min), ultrafiltration (0.45 μm) and purified by sequential amylose and Ni–NTA affinity chromatography. Glucose (20% w/v) was used for elution of MBP-fusion proteins to enable reuse of MBP in subsequent purification steps. Purified MBP–procoil–CTA2/CTB complexes were dialyzed into HEPES buffer (50 mM HEPES, 150 mM NaCl, pH 7.4). For PaP, equimolar mixtures of MBP–PaP.1 and MBP–PaP.2 were combined and further purified by size-exclusion chromatography to isolate the dimeric species. The dialyzed or purified protein was then concentrated to 50 μM, and the MBP was removed by applying 5 mol % of MBP-His-TEV protease or 6 mol % factor Xa protease overnight at 25 °C. The resulting mixtures were passed through amylose and Ni–NTA columns to separate the cleaved MBP and protease from the desired procoil–CTA2/CTB complexes.

### Size Exclusion Chromatography (SEC)

SEC was performed at 4 °C using the Bio-Rad FPLC system. Typically, 50 μL of 30 μM protein was loaded onto either a Superdex 200 (10/300 or 16/60) or Superdex 75 (16/60) column, depending on the molecular weight of the target complex. Elution was carried out isocratically in HEPES buffer (50 mM HEPES, 150 mM NaCl, pH 7.4) at flow rates of 0.4–1 mL/min. Elution profiles were monitored by UV absorbance at 280 nm, with additional channels for 495 or 647 nm where fluorescently labeled proteins were analyzed.

### Size Exclusion Chromatography with Multiangle Light Scattering (SEC-MALS)

SEC-MALS was used to determine the oligomeric state of selected MBP–procoil–CTA2 constructs. Samples (35 μL, 3 mg/mL) were injected onto a Superose 6 Increase 5/150 column and eluted in HEPES buffer (50 mM HEPES, 200 mM NaCl, pH 7.5) at 0.2 mL/min. Data were collected using UV, refractive index, and multiangle light scattering detectors and analyzed with Astra 6.2 software (Wyatt Technology). MBP-AnP2 eluted as a dimeric species, and MBP-PaP1 as a monomer, consistent with their theoretical molecular weights (109 987 Da and 112 822 Da, respectively).

### Mass Photometry

The oligomeric states of AnP1, AnP2, and PaP were further assessed by mass photometry (Refeyn OneMP) over a concentration range of 20–80 nM. Protein samples were diluted immediately before measurement in HEPES buffer (50 mM HEPES, 150 mM NaCl, pH 7.4). Data were acquired and processed using Refeyn AcquireMP and DiscoverMP software. Mass distributions were calibrated against BSA standards and fitted with Gaussian functions to determine average molecular masses.

### Atomic Force Microscopy (AFM)

AFM was used to image AnP1, AnP2, and PaP deposited on mica in aqueous buffer. One μL of 100 nM dimer protein solution was added to 150 μL of adsorption buffer (150 mM NaCl, 50 mM HEPES, pH 7.4) on freshly cleaved mica and incubated for 60 s to allow surface binding. The sample was then gently rinsed by fluid exchange with the same buffer. Subsequently, 10 μL of 100 mM NiCl_2_ was added to the imaging buffer to achieve a final concentration of 6.25 mM NiCl_2_. All AFM imaging was performed at room temperature with the dimers fully hydrated in solution. Observations were carried out in PeakForce Tapping mode using a Dimension FastScan Bio instrument with sharp (1 nm nominal tip radius) PeakForce HIRS-F-B and PeakForce HIRS-SSB probes (Bruker) with nominal spring constants of 0.12 N/m. AFM image processing and analysis was performed using the NanoLocz software package.^46^ Particle detection was carried out using the peaks workflow with Gaussian filtering applied to ensure one peak per monomer. The resulting x–y coordinates were used to calculate pairwise distances between all neighboring particles.

### MD Simulation

Explicit-solvent atomistic-detail simulations were performed using the Amber 20 software suite, and employed the Amber-ff14SB force field. All three protein complexes (AnP1, AnP2, PaP) were first solvated in a cubic box of TIP3P water containing 150 mM NaCl plus additional Na/Cl ions for balancing net charge on the proteins. The minimum separation between the protein surface and the water box’s edge was set at 12 Å. Each simulation was divided into three phases. First, potential energy conflicts within the solvent environment were reduced by applying 2500 steps of steepest descent minimization, followed by up to 2500 steps of conjugate gradient minimization. All peptide backbone atoms were tightly constrained using a force of 2.0 kcal/mol/Å^2^. In the second phase, the system was heated from 10.0 to 303.15 K (30 °C) over 25 ps. A Langevin thermostat with a friction coefficient of 0.2 ps^–1^ controlled the temperature, and the strength of the constraints on the protein was decreased to 0.1 kcal/mol/Å^2^, allowing some relaxation of the backbone. Lastly, the positional restraints were removed entirely, and the simulation was switched to an isotropic NPT regime, kept at 1.0 bar with a Monte Carlo barostat. Here the simulation time steps were changed from 0.001 to 0.004 ps, which was possible thanks to hydrogen mass repartitioning by ParmEd, and atom positions were recorded every 0.5 ns. The production phase lasted between 587 and 1040 ns, of which the first 100 ns were considered extended equilibration and always excluded from subsequent analysis. The simulation trajectories were analyzed using AmberTools and in-house Python scripts. Throughout all phases, the Particle-Mesh Ewald method was employed to calculate long-range electrostatic interactions, with a grid spacing of 0.16 nm and a cutoff of 1.2 nm, and bonds involving hydrogen atoms were constrained using the SHAKE algorithm.

### Flow Cytometry

For flow cytometry, 1 × 10^5^ Jurkat T cells were seeded per well in a U-bottom 96 well plate (Sarstedt). Cells were incubated with increasing concentrations (0.25–40 nM CTB pentamers) of AF488-labeled protein for 30 min at 4 °C in the dark. PBS-treated cells served as negative controls. After incubation, cells were washed twice with FACS buffer (PBS supplemented with 3% FCS v/v) and resuspended in the same buffer on ice. Fluorescence intensity was recorded on a Beckman Coulter Gallios cytometer, and data were analyzed with FlowJo v10.5.3. Binding curves were fitted to a Hill equation in OriginPro to calculate EC_50_ values.

### Lipid Mixing Assay

Lipid mixing was assessed using a Förster resonance energy transfer (FRET) assay with NBD- and rhodamine-labeled phosphatidylethanolamine (PE-NBD, PE-Rho). Large unilamellar vesicles (LUVs, 800 nm) were prepared by thin-film hydration, freeze–thaw cycling, and extrusion in HEPES buffer (50 mM HEPES, 150 mM NaCl, pH 7.4). LUVs containing the FRET pair (FRET-LUVs) comprised DOPC:DOPE:cholesterol:GM1:PE-NBD:PE-Rho (47:24:24:5:0.25:0.25). LUVs without the FRET pair (Blank-LUVs) comprised DOPC:DOPE:cholesterol:GM1 (48:24:24:5). Protein samples were added to a 1:4 mixture of FRET-LUVs and blank-LUVs to give a final lipid concentration of 133 μM lipid and up to 4 μM CTB in small volume 384-well plates (Greiner Bio-One). Triton X-100 (0.2% v/v) was used as a positive control for complete fusion, similar to the method used by Struck et al.,^37^ and vesicles incubated without protein served as negative controls. Fluorescence emission spectra (500–630 nm) were recorded upon excitation at 470 nm. The FRET ratio *R* = *I*_590_/*I*_530_ was calculated from the intensities of rhodamine and NBD, respectively. Lipid mixing was expressed as a percentage relative to the Triton control:

$$\%Lipid\;mixing=\left(R_{n}-R_{0}\right)\times 100/R_{full\text{\_}fusion}-R_{0}$$

where *R*_*n*_ is the FRET ratio at time *n* min, *R*_0_ is the initial value, and *R*_*full_fusion*_ is the Triton control. Data represent three independent technical replicates. Rates of lipid mixing were determined by global fitting of the time courses in OriginPro, and plotted as a function of protein concentration.

### GUVs Preparation and Imaging

GUVs were prepared by electroformation following Madl et al.^47^ Lipid mixtures (0.5 mg/mL in chloroform) contained 30 mol % cholesterol, 64.7 mol % DOPC, 5 mol % GM1, and 0.3 mol % DOPE-Atto647N (for fluorescent GUVs). Nonfluorescent GUVs were prepared identically but without DOPE-Atto647N and with 65 mol % DOPC. Lipid films were deposited on indium–tin–oxide (ITO) coated slides, dried under vacuum, and rehydrated in 330 mOsm sucrose solution. Electroformation was carried out for 140 min at 10 Hz, 1.1 V. GUVs were collected immediately, stored on ice, and used on the same day.

Observation chambers were assembled by attaching 8 × 8 mm cloning cylinders to glass coverslips. Chambers were precoated with 1 mg/mL β-casein for 30 min and washed with 330 mOsm DPBS to prevent nonspecific vesicle adhesion.

Confocal imaging was performed on a Nikon A1R laser scanning microscope with a 60× oil immersion objective. Excitation was provided at 405, 488, and 640 nm. For imaging, 30–40 μL of GUV suspension was diluted in DPBS to 200 μL total volume; when mixing two GUV populations, 15–20 μL of each was used. Proteins (AnP1, AnP2, or PaP) were added to a final dimer concentration of 200 nM (i.e., 400 nM pentamer). Chambers were covered with glass to minimize evaporation, and time-lapse images were collected every 30 s for up to 2 h from multiple positions chosen based on GUV density.

### Quartz Crystal Microbalance with Dissipation Monitoring (QCM-D)

QCM-D experiments were carried out using silica-coated QCM-D sensors (QSX303, Biolin Scientific) on a Q-Sense E4 system at 23 °C in flow mode (20 μL/min) with HBS buffer (10 mM HEPES, 150 mM NaCl, pH 7.4). Sensors were cleaned by 30 min UV/ozone treatment prior to use. Frequency and dissipation shifts were recorded at multiple overtones *j* = 3, 5, 7, 9, 11, and 13 (corresponding to resonance frequencies of approximately 15, 25, 35, 45, 55, and 65 MHz) and the fundamental resonance frequency (∼5 MHz). Dissipation shifts, Δ*D*, and normalized frequency shifts, Δ*F* = Δ*f*_*i*_/*i*, for *i* = 5 are presented. All other overtones gave qualitatively similar results.

Supported lipid bilayers (SLBs) were formed by flowing SUVs (50 μg/mL, DOPC with or without 5 mol % GM1) over the sensors. SLB formation was confirmed by Δ*F* ≈ −26 Hz and Δ*D* < 0.5 × 10^–6^. CTB dimer constructs (400 nM CTB pentamer equivalent) were then introduced, followed by a second SUV injection to test for binding to membrane-facing CTB sites. Wash steps with HBS buffer were included between each addition.

The thickness of SLBs and dense protein monolayers was estimated using a modified Sauerbrey equation, $h=\frac{-C\times \Delta F}{\rho }$ , with a mass sensitivity constant *C* = 18.0 ng/cm^2^/Hz, and a density ρ = 1.0 g/cm^3^ (for lipids) and ρ ≈ 1.1 g/cm^3^ (for a well-solvated protein layer, to a good approximation), as previously established.^48^ Analysis was performed using QSoft401 version 2.7.3.883.

## Supplementary Material





## Data Availability

This manuscript was previously made available as a preprint: Dai, W.; Kempmann, E.; Rosato, F.; Nikolova, M.; Siukstaite, L.; Kamiński, T.P.; Booth, A.; Ishmael, M.S.K.; Wang, C.; Heath, G.R.; Beales, P.A.; Richter, R.P.; Römer, W.; Webb, M.E.; Turnbull, W.B. Engineered Coiled-Coils Convert Cholera Toxin B-Pentamers into Programmable Membrane Fusogens. 2025, 2025-mnsj9. *ChemRxiv*. 10.26434/chemrxiv-2025-mnsj9 (accessed April 27, 2026). Data associated with this paper is available at 10.5518/1861.

## Notes

Coiled-coil sequences and protein sequences for AnP1, AnP2 and PaP; ISAMBARD modeling for the coiled coils; molecular simulation results for AnP1, AnP2 and PaP; kinetic data for the lipid mixing assay; negative control of GUVs without the presence of any protein; QCM-D experiments; flow cytometry data; negative control of Jurkat cells and GUVs without the presence of protein (PDF)
Supporting Videos SV1–SV8: Video SV1: GUV cross-linking and fusion induced by AnP2; Video SV2: GUV cross-linking and fusion induced by AnP1; Video SV3: GUV cross-linking and fusion induced by PaP; Video SV4: Jurkat cells and GUVs without protein; Video SV5: CTA2/CTB protein with Jurkat cells and GUVs; Video SV6 and SV7: Cross-linking of Jurkat cells and GUVs by AnP1; Video SV8: Cross-linking of Jurkat cells and GUVs by AnP2 (ZIP)

## References

[1] Wassarman, P. M. ; Litscher, E. S. Mammalian Fertilization Is Dependent on Multiple Membrane Fusion Events. In Cell Fusion, Chen, E. H. , Eds.; Humana Press: Totowa, NJ, 2008; pp. 99–113. 10.1007/978-1-59745-250-2_6 18979240

[2] Jahn R. (1998). Synaptic Transmission:
Two Players Team up for a New Tune. Curr. Biol..

[3] Martens S., McMahon H. T. (2008). Mechanisms of Membrane
Fusion: Disparate Players and
Common Principles. Nature Rev. Mol. Cell Biol..

[4] Ni F., Chen X., Shen J., Wang Q. (2014). Structural Insights
into the Membrane Fusion Mechanism Mediated by Influenza Virus Hemagglutinin. Biochemistry.

[5] Weber T., Zemelman B. V., McNew J. A., Westermann B., Gmachl M., Parlati F., Söllner T. H., Rothman J. E. (1998). Snarepins: Minimal Machinery for Membrane Fusion. Cell.

[6] Südhof T. C., Rothman J. E. (2009). Membrane Fusion: Grappling with Snare and Sm Proteins. Science.

[7] Crick F. H. C. (1953). The
Packing of Α-Helices: Simple Coiled-Coils. Acta Crystallogr..

[8] Chen Y. A., Scheller R. H. (2001). Snare-Mediated Membrane Fusion. Nature Rev. Mol. Cell Biol..

[9] Kashiwada A., Tsuboi M., Takamura N., Brandenburg E., Matsuda K., Koksch B. (2011). Design and Characterization of Endosomal-Ph-Responsive
Coiled Coils for Constructing an Artificial Membrane Fusion System. Chem.—Eur. J..

[10] Gong Y., Ma M., Luo Y., Bong D. (2008). Functional Determinants of a Synthetic
Vesicle Fusion System. J. Am. Chem. Soc..

[11] Zeng Y., Shen M., Singhal A., Sevink G. J. A., Crone N., Boyle A. L., Kros A. (2023). Enhanced Liposomal
Drug Delivery
Via Membrane Fusion Triggered by Dimeric Coiled-Coil Peptides. Small.

[12] Ma M., Bong D. (2013). Controlled Fusion of Synthetic Lipid Membrane Vesicles. Acc. Chem. Res..

[13] Chan Y. H. M., van Lengerich B., Boxer S. G. (2009). Effects of Linker
Sequences on Vesicle Fusion Mediated by Lipid-Anchored DNA Oligonucleotides. Proc. Natl. Acad. Sci. U. S. A..

[14] Jumeaux C., Wahlsten O., Block S., Kim E., Chandrawati R., Howes P. D., Höök F., Stevens M. M. (2018). Microrna Detection
by DNA-Mediated Liposome Fusion. ChemBiochem.

[15] Marsden H. R., Tomatsu I., Kros A. (2011). Model Systems
for Membrane Fusion. Chem. Soc. Rev..

[16] Zheng T., Voskuhl J., Versluis F., Zope H. R., Tomatsu I., Marsden H. R., Kros A. (2013). Controlling the Rate of Coiled Coil
Driven Membrane Fusion. Chem. Commun..

[17] Daudey G. A., Zope H. R., Voskuhl J., Kros A., Boyle A. L. (2017). Membrane-Fusogen
Distance Is Critical for Efficient Coiled-Coil-Peptide-Mediated Liposome
Fusion. Langmuir.

[18] Versluis F., Dominguez J., Voskuhl J., Kros A. (2013). Coiled-Coil Driven
Membrane Fusion: Zipper-Like Vs. Non-Zipper-Like Peptide Orientation. Faraday Discuss.

[19] Koukalová A., Pokorná Š., Boyle A. L., Lopez
Mora N., Kros A., Hof M., Šachl R. (2018). Distinct Roles
of Snare-Mimicking Lipopeptides During Initial Steps of Membrane Fusion. Nanoscale.

[20] Daudey G. A., Shen M., Singhal A., van der Est P., Sevink G. J. A., Boyle A. L., Kros A. (2021). Liposome Fusion with
Orthogonal Coiled Coil Peptides as Fusogens: The Efficacy of Roleplaying
Peptides. Chem. Sci..

[21] Aschmann D., Knol R. A., Kros A. (2024). Lipid-Based
Nanoparticle Functionalization
with Coiled-Coil Peptides for in Vitro and in Vivo Drug Delivery. Acc. Chem. Res..

[22] Balenzano G., Eczacioglu N., Denora N., Bernkop-Schnürch A. (2025). Fusogenic
Lipid Nanocarriers: Nature-Inspired Design for Advanced Drug Delivery
Systems. Adv. Colloid Interface Sci..

[23] Wehrum S., Siukstaite L., Williamson D. J., Branson T. R., Sych T., Madl J., Wildsmith G. C., Dai W., Kempmann E., Ross J. F., Thomsen M., Webb M. E., Römer W., Turnbull W. B. (2022). Membrane Fusion Mediated by Non-Covalent Binding of
Re-Engineered Cholera Toxin Assemblies to Glycolipids. ACS Syn. Biol..

[24] Beddoe T., Paton A. W., Le Nours J., Rossjohn J., Paton J. C. (2010). Structure,
Biological Functions and Applications of the Ab5 Toxins. Trends Biochem. Sci..

[25] Turnbull W. B., Precious B. L., Homans S. W. (2004). Dissecting the Cholera
Toxin-Ganglioside
Gm1 Interaction by Isothermal Titration Calorimetry. J. Am. Chem. Soc..

[26] Shi J., Yang T., Kataoka S., Zhang Y., Diaz A. J., Cremer P. S. (2007). Gm1 Clustering Inhibits Cholera Toxin Binding in Supported
Phospholipid Membranes. J. Am. Chem. Soc..

[27] Montesano R., Roth J., Robert A., Orci L. (1982). Non-Coated Membrane
Invaginations Are Involved in Binding and Internalization of Cholera
and Tetanus Toxins. Nature.

[28] Ewers H., Römer W., Smith A. E., Bacia K., Dmitrieff S., Chai W., Mancini R., Kartenbeck J., Chambon V., Berland L., Oppenheim A., Schwarzmann G., Feizi T., Schwille P., Sens P., Helenius A., Johannes L. (2010). Gm1 Structure Determines Sv40-Induced
Membrane Invagination and Infection. Nat. Cell
Biol..

[29] Merritt E. A., Kuhn P., Sarfaty S., Erbe J. L., Holmes R. K., Hol W. G. J. (1998). The 1.25 Å
Resolution Refinement of the Cholera
Toxin B-Pentamer: Evidence of Peptide Backbone Strain at the Receptor-Binding
Site. J. Mol. Biol..

[30] Dertzbaugh M. T., Cox L. M. (1998). The Affinity of
Cholera Toxin for Ni^2+^ Ion. Protein
Eng., Des. Sel..

[31] Thompson K. E., Bashor C. J., Lim W. A., Keating A. E. (2012). Synzip
Protein Interaction
Toolbox: In Vitro and in Vivo Specifications of Heterospecific Coiled-Coil
Interaction Domains. ACS Syn. Biol..

[32] Anderson G. P., Shriver-Lake L. C., Liu J. L., Goldman E. R. (2018). Orthogonal Synthetic
Zippers as Protein Scaffolds. ACS Omega.

[33] Gurnon D. G., Whitaker J. A., Oakley M. G. (2003). Design
and Characterization of a
Homodimeric Antiparallel Coiled Coil. J. Am.
Chem. Soc..

[34] Patterson D. P., Su M., Franzmann T. M., Sciore A., Skiniotis G., Marsh E. N. G. (2014). Characterization of a Highly Flexible Self-Assembling
Protein System Designed to Form Nanocages. Protein
Sci..

[35] Smith T. J., Tardu M., Khatri H. R., Koutmou K. S. (2022). Mrna and Trna Modification
States Influence Ribosome Speed and Frame Maintenance During Poly­(Lysine)
Peptide Synthesis. J. Biol. Chem..

[36] Wood C. W., Heal J. W., Thomson A. R., Bartlett G. J., Ibarra A. Á., Brady R. L., Sessions R. B., Woolfson D. N. (2017). Isambard: An Open-Source
Computational Environment for Biomolecular Analysis, Modelling and
Design. Bioinformatics.

[37] Struck D. K., Hoekstra D., Pagano R. E. (1981). Use of
Resonance Energy Transfer
to Monitor Membrane Fusion. Biochemistry.

[38] Amjad O. A., Mognetti B. M., Cicuta P., Di Michele L. (2017). Membrane Adhesion
through Bridging by Multimeric Ligands. Langmuir.

[39] Richter R. P., Bérat R., Brisson A. R. (2006). Formation of Solid-Supported Lipid
Bilayers: An Integrated View. Langmuir.

[40] Arnott Z. L. P., Morgan H. E., Hollingsworth K., Stevenson C. M. E., Collins L. J., Tamasanu A., Machin D. C., Dolan J. P., Kamiński T. P., Wildsmith G. C. (2024). Quantitative N- or C-Terminal
Labelling of Proteins with Unactivated Peptides by Use of Sortases
and a D-Aminopeptidase. Angew. Chem., Int. Ed.

[41] Mou J., Yang J., Shao Z. (1995). Atomic Force Microscopy of Cholera
Toxin B-Oligomers Bound to Bilayers of Biologically Relevant Lipids. J. Mol. Biol..

[42] Briand E., Zäch M., Svedhem S., Kasemo B., Petronis S. (2010). Combined Qcm-D
and Eis Study of Supported Lipid Bilayer Formation and Interaction
with Pore-Forming Peptides. Analyst.

[43] Lian T., Ho R. J. Y. (1997). Cholera Toxin
B-Mediated Targeting of Lipid Vesicles
Containing Ganglioside Gm1 to Mucosal Epithelial Cells. Pharm. Res..

[44] Haigh J. L., Williamson D. J., Poole E., Guo Y., Zhou D., Webb M. E., Deuchars S. A., Deuchars J., Turnbull W. B. (2020). A Versatile
Cholera Toxin Conjugate for Neuronal Targeting and Tracing. Chem. Commun..

[45] Balmforth M. R., Haigh J., Kumar V., Dai W., Tiede C., Tomlinson D. C., Deuchars J., Webb M. E., Turnbull W. B. (2021). Piggybacking
on the Cholera Toxin: Identification of a CTB-Binding Protein as an
Approach for Targeted Delivery of Proteins to Motor Neurons. Bioconjugate Chem..

[46] Heath G. R., Micklethwaite E., Storer T. M. (2024). Nanolocz: Image Analysis Platform
for AFM, High-Speed AFM, and Localization AFM. Small Methods.

[47] Madl, J. ; Villringer, S. ; Römer, W. Delving into Lipid-Driven Endocytic Mechanisms Using Biomimetic Membranes. In Chemical and Synthetic Approaches in Membrane Biology, Shukla, A. , Eds.; Humana Press: New York, NY, 2016; pp. 17–36.

[48] Reviakine I., Johannsmann D., Richter R. P. (2011). Hearing What You Cannot See and Visualizing
What You Hear: Interpreting Quartz Crystal Microbalance Data from
Solvated Interfaces. Anal. Chem..

