# Supplementary material for: Engineered Coiled-Coils Convert Cholera Toxin B‑Pentamers into Programmable Membrane Fusogens
Source: ACS Nano. 2026 May 13;20(20):14645–58. doi: 10.1021/acsnano.6c01578 (PMC13218038; doi:10.1021/acsnano.6c01578)
Supplement: Supplementary file 1 [file nn6c01578_si_001.pdf]

Supporting Information

Engineered coiled-coils convert cholera toxin B-pentamers into programmable membrane fusogens

Wenyue Dai[1,2], Erik Kempmann[3,4],‡ Francesca Rosato[3,4],‡ Maria Nikolova[2,5], Lina Siukstaite[3,4], Tomasz P. Kamiński[1,2], Andrew Booth[1], Maryam S. K. Ishmael[1,2], Chunyue Wang[2,5,6,7], George R. Heath[2,5,6,7], Paul A. Beales[1,2,7], Ralf P. Richter[2,5,6,7], Winfried Römer[3,4], Michael E. Webb[1,2*], W. Bruce Turnbull,[1,2*†]

KEYWORDS: membrane fusion; programmable biointerfaces; coiled-coil protein engineering; lectin–glycolipid interactions; synthetic fusogens; nanobiotechnology

Affiliations:

1 School of Chemistry, University of Leeds, Leeds LS2 9JT, UK

2 Astbury Centre for Structural Molecular Biology, University of Leeds, Leeds LS2 9JT, UK

3 Faculty of Biology, Synthetic Biology of Signalling Processes lab, University of Freiburg, 79104 Freiburg, Germany

4 Signalling Research Centres BIOSS and CIBSS, University of Freiburg, 79104 Freiburg, Germany

5 School of Biomedical Sciences, University of Leeds, Leeds LS2 9JT, UK

6 School of Physics and Astronomy, University of Leeds, Leeds LS2 9JT, UK

7 Bragg Centre for Materials Research, University of Leeds, Leeds LS2 9JT, UK

Table of Contents

SECTION 1 Protein Sequences 2

SECTION 2 Isambard And Coiled-Coil Modelling 4

SECTION 3 Molecular Simulations 10

SECTION 4 Proteolysis of MBP-AnP2 13

SECTION 5 Lipid mixing assay 14

SECTION 6 Negative Control of GUVs 16

SECTION 7 QCM-D 17

SECTION 8 Atomic Force Microscopy 20

SECTION 9 Flow Cytometry 21

SECTION 10 Negative control of jurkat cell and guvs 22

REFERENCES 24

A range of coiled-coil designs (both parallel and antiparallel, homodimeric and heterodimeric), including published sequences and derivatives thereof,[1-5] were cloned into the pTRBAB5-G1S expression plasmid between a maltose-binding protein (MBP) affinity tag and the CTA2 sequence for coexpression with CTB.[6] From these constructs, we selected the following sequences (Figures S1-1, S1-2) that demonstrated optimal expression and stability in forming MBP-procoil-CTA/CTB dimers.

PaP – parallel heterodimeric coiled-coil sequences are identical to the Keating group SYNZIP 1-2 pair.[2]

AnP1 – antiparallel homodimeric coiled-coil was derived from an Oakley group sequence.[4] Non-interacting lysine and tryptophan residues at the *f* positions in the heptad sequence (and lysine at position "2b" that did not appear essential for the coiled-coil formation) were mutated to alanine or glutamine to improve expression of the AnP1 construct.

AnP2 - antiparallel homodimeric coiled-coil was derived from a Marsh group sequence.[5] Non-interacting lysine and tryptophan residues at the *f* positions in the heptad sequence were mutated to glutamate to improve expression of the AnP2 construct. While we had chosen this sequence to become part of a heterodimeric pair, we found the AnP2 construct formed homodimers with similar stability to the PaP and AnP2 constructs (manuscript Figure 2).

Modelling (Supporting information section 2) confirmed that the AnP1 and AnP2 sequences should preferentially form antiparallel coiled coils like their parent sequences.

**Heptad reference positions**	*fg*	*abcdefg*	*abcdefg*	*abcdefg*	*abcdefg*	*abcdefg*	*abcdefg*	*abcdefg*
**PaP.1 pro-coil** (SYNZIP 1 - ref 2)	NL	VAQLENE	VASLENE	NETLKKK	NLHKKDL	IAYLEKE	IANLRKK	IEEP
**Pap.2 pro-coil** (SYNZIP 2 - ref 2)	AR	NAYLRKK	IARLKKD	NLQLERD	EQNLEKI	IANLRDE	IARLENE	VASHEQ
**AnP1 pro-coil**		QLEQE	LAQLEAE	LQAIEQQ	LAQLQAK	AQARKQK	LAQLKAK	LQA
AnP1 was derived from a sequence in ref 4		QLEKE	LKQLEKE	LQAIEKQ	LAQLQWK	AQARKKK	LAQLKKK	LQA
**AnP2 pro-coil**		QLEKE	LAQLKEK	LQAIEKE	LAQLKEK	AQALKEK	LAQLKEK	LQ
AnP2 was derived from a sequence in ref 5		QLEKE	LAQLKKK	LQAIEKE	LAQLKWK	AQALKKK	LAQLKKK	LQ
**Heptad reference positions**	*fg*	*abcdefg*	*abcdefg*	*abcdefg*	*abcdefg*	*abcdefg*	*abcdefg*	*abcdefg*

Figure S1-1. Pro-coil sequences selected for constructing CTB dimers coiled-coils. Heptad reference positions *c* and *d* form the hydrophobic core of the coiled-coil, while positions *e* and *g* form stabilising salt bridges. Position *f* sits furthest from the hydrophobic core on the outside of the coiled-coil.

MBP-AnP1-CTA2

MBP-linker	KIEEGKLVIWINGDKGYNGLAEVGKKFEKDTGIKVTVEHPDKLEEKFPQVAATGDGPD<br>IIFWAHDRFGGYAQSGLLAEITPDKAFQDKLYPFTWDAVRYNGKLIAYPIAVEALSLI
Factor Xa site<br>TEV site<br>GS linker<br>N-cap<br>AnP1<br>Proline break<br>full-length CTA2	YNKDLLPNPPKTWEEIPALDKELKAKGKSALMFNLQEPYFTWPLIAADGGYAFKYENG<br>KYDIKDVGVDNAGAKAGLTFLVDLIKNKHMNADTDYSIAEAAFNKGETAMTINGPWAW<br>SNIDTSKVNYGVTVLPTFKGQPSKPFVGVLSAGINAASPNKELAKEFLENYLLTDEGL<br>EAVNKDKPLGAVALKSYEEELVKDPRIAATMENAQKGEIMPNIPQMSAFWYAVRTAVI<br>NAASGRQTVDEALKDAQTNSSSNNNNNNNNNNNLGIEGRISHMGSENLYFQGVGGSGSG<br>SGSGTPAEQLEQELAQLEAELQAIEQQLAQLQAKAQARKQKLAQLKAKLQAPDEKTQS<br>HGVKFLDEYQSKVKRQIFSGYQSDIDTHNRIKDEL

MBP-AnP2-sCTA2

Factor Xa site<br>TEV site<br>AnP2<br>short CTA2 (sCTA2)	......GIEGRISHMGSENLYFQSVGQLEKELAQLKEKLQAIEKELAQLKEKAQALKEKLAQ<br>LKEKLQQSKVKRQIFSGYQSDIDTHNRIKDEL

MBP-PaP.1-CTA2

Factor Xa site<br>TEV site<br>PaP.1<br>Proline break<br>full-length CTA2	......GIEGRISHMGSENLYFQSVGNLVAQLENEVASLENENETLKKKNLHKKDLIAYLEK<br>EIANLRKKIEEPDEKTQSHGVKFLDEYQSKVKRQIFSGYQSDIDTHNRIKDEL

MBP-PaP.2-CTA2

Factor Xa site<br>TEV site<br>PaP.2<br>Proline break<br>full-length CTA2	......GIEGRISHMGSENLYFQSVGARNAYLRKKIARLKKDNLQLERDEQNLEKIIANLRD<br>EIARLENEVASHEQPDEKTQSHGVKFLDEYQSKVKRQIFSGYQSDIDTHNRIKDEL

CTB

APQNITDLCAEYHNTQIYTLNDKIFSYTESLAGKREMAIITFKNGAIFQVEVPGSQ
HIDSQKKAIERMKDTLRIAYLTEAKVEKLCVWNNKTPHAIAAISMAN

Figure S1-2. The protein sequences for the MBP-procoil-CTA2/CTB fusion constructs. For MBP-AnP1, MBP-PaP.1, and MBP-PaP.2, TEV protease cleavage was employed post-expression to separate MBP from the fusion protein. For MBP-AnP2, cleavage was facilitated by Factor Xa protease to achieve the same separation.

To assess orientation preferences of selected coiled-coil, we used ISAMBARD, a modelling package designed for coiled-coil structures using the BUDE forcefield (Bristol University Docking Engine Force Field).[7] ISAMBARD allowed grid-based coarse screening of parameters including coiled-coil radius, pitch, PhiCa, and Zshift, followed by a parameter optimization process using the genetic algorithm embedded in ISAMBARD (Figure S2-1).

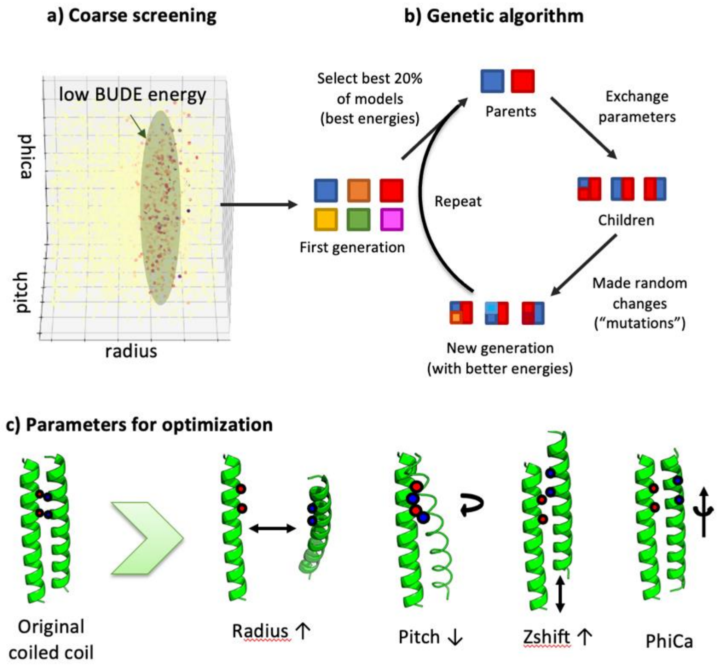

Figure S2-1. Schematic flowchart to generate coiled-coil models. (a) An initial coarse screen of parameters is followed by use of (b) a genetic algorithm to optimise the best combination of parameters set for the given-coiled coil sequence. (c) Parameters to be optimized include Radius, Pitch, Z-shift and PhiCa.

	Input sequence	Orientation	Radius	Pitch	PhiCA	Zshift
**PaP**	PaP.1 LVAQLENEVASLENENETLKKKN LHKKDLIAYLEKEIANLRKKIEE PaP.2 RNAYLRKKIARLKKDNLQLERDE QNLEKIIANLRDEIARLENEVAS	**Parallel**	5.5 ± 2.5 Å	200 ± 60 Å	283 ± 40°	0 ± 20 Å
		**Antiparallel**	5.5 ± 2.5 Å	200 ± 60 Å	283 ± 40°	0 ± 20 Å
**AnP1**	PAEQLEQELAQLEAELQAIEQQL AQLQAKAQARKQKLAQLKAKLQA	**Parallel**	5.5 ± 2.5 Å	200 ± 60 Å	283 ± 40°	0 ± 20 Å
		**Antiparallel**	5.5 ± 2.5 Å	200 ± 60 Å	283 ± 200°	0 ± 10 Å
**AnP2**	SENLYFQSVGQLEKELAQLKEKL QAIEKELAQLKEKAQALKEKLAQ LKEKLQ	**Parallel**	5.5 ± 2.5 Å	200 ± 60 Å	283 ± 100°	0 ± 30 Å
		**Antiparallel**	5.5 ± 2.5 Å	200 ± 60 Å	283 ± 100°	0 ± 30 Å

Figure S2-2. Protein sequences and initial parameters used to construct the coiled-coil structural models for PaP (PaP.1 and PaP.2 as parallel heterodimer), AnP1 and AnP2.

Coarse Screening of Parameters

Starting from the parameter ranges in Figure S2-2, grid-based sampling of parameters (Radius, Pitch, PhiCa, Z-shift) identified low-energy regions for each sequence preset to be either parallel or antiparallel. The registration number for the coiled coil, PhiCA, with values for positions “a” through “g” designated as 0, 102.8, 205.6, 308.4, 51.4, 154.2, and 257, respectively, and a default PhiCA value of 283, corresponding to position “g”. Screening was conducted three times in parallel, producing 3000 parameter combinations. The combined results were plotted on two 3D scatter plots using Radius, Pitch, and PhiCA as axes (Figure S2-3), with colours indicating energy score.

Genetic Algorithm Optimization

The top 20% scoring models were refined using ISAMBARD’s genetic algorithm, narrowing to 400 candidates and selecting the best model based on RMSD as shown in the energy funnel plots (Figure S2-4). The selected model was further used to initiate another 50 iterations of optimization (Figure S2-5). This selection process was performed three times in parallel, allowing calculation of the average BUDE interaction energy and standard deviation. Ultimately, the refined model with the lowest BUDE interaction energy was chosen to fuse with the CTA2/CTB model (PDB: 1XTC) for molecular simulation (Supporting information section 3).

Orientation Insights

ISAMBARD predicted that both AnP1 and Anp2 as having antiparallel orientations as expected. In the case of AnP2, ISAMBARD’s prediction indicated that the TEV cleavage sequence could be involved in extending the coiled-coil interface which is consistent with the inefficient cutting of this sequence by TEV protease (Figure S4-1). For PaP, the ISAMBARD predictions were ambiguous, despite X-ray crystallography evidence that this sequence has a parallel orientation,[2] which might be a consequence of limitations of BUDE in modelling hydrophobic interface solvation and entropy.[8] Modelling the PaP coiled-coils in AlphaFold3 concluded that it would have a parallel orientation (Figure S2-6). Similarly, AnP1 and AnP2 were predicted to be antiparallel, albeit with lower confidence for AnP2.

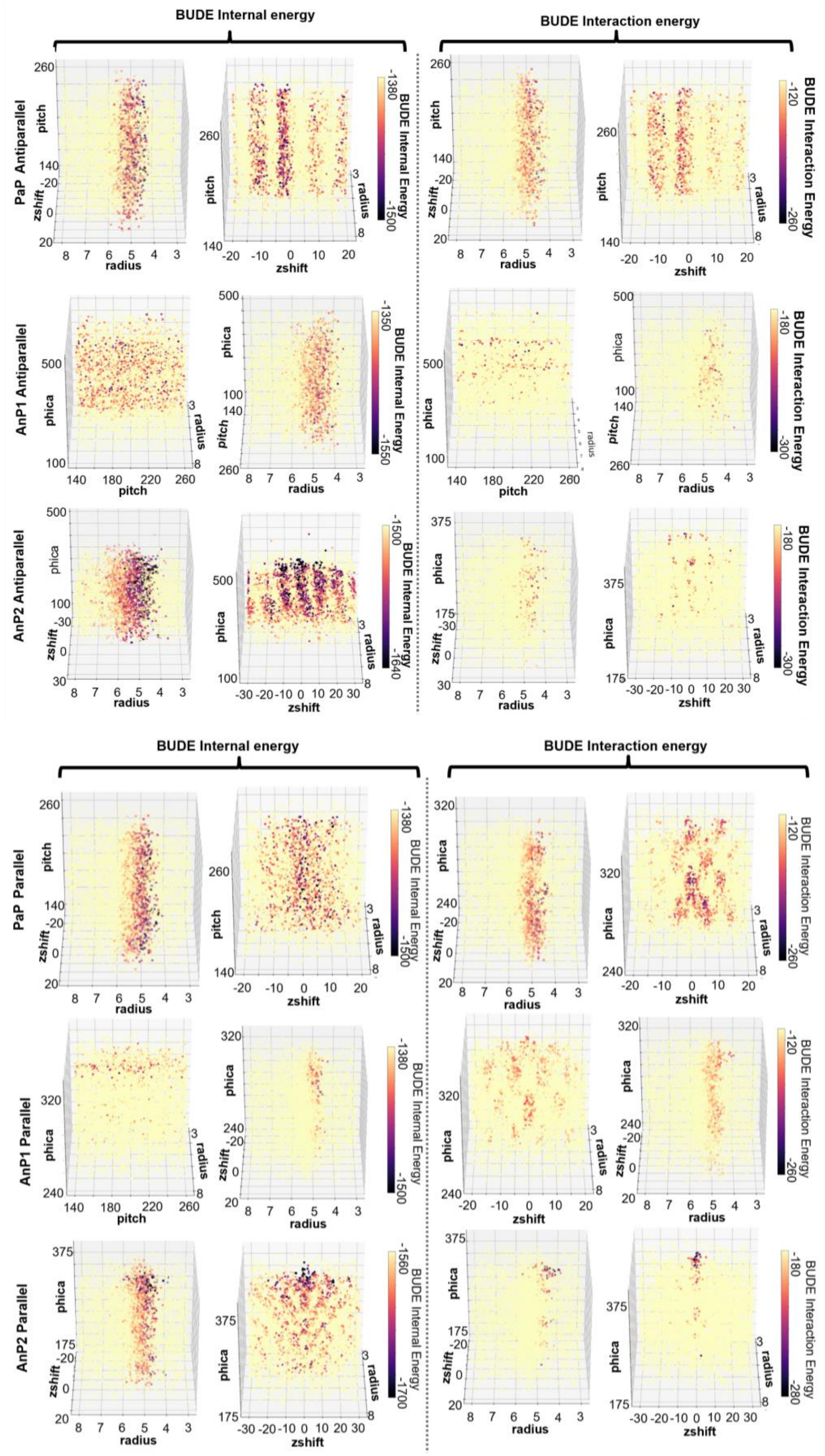

Figure S2-3. 3D scatter plots showing coarse screening results for the selected coiled coil sequence. Axes represent **Z-shift (x)**, **Pitch (y)**, and **Radius (z)**. Points are colored by BUDE energy scores, with darker purple indicating low-energy regions. Selected parameter sets within these regions are highlighted for further refinement.

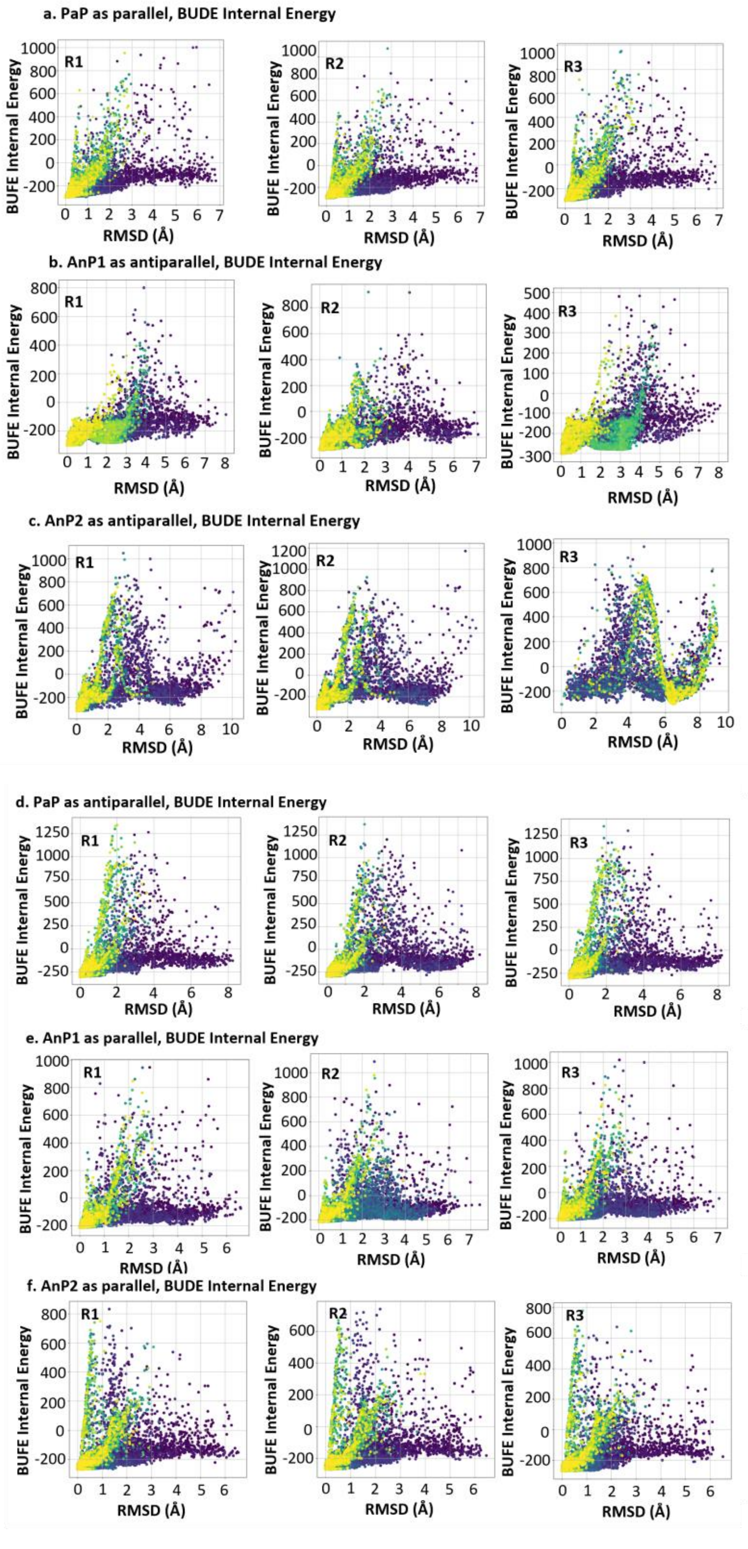

Figure S2-4. Energy funnel plots from three rounds of genetic algorithm optimization for coiled-coil models in both parallel and antiparallel orientations. Each plot shows **RMSD (Å)** to the best-scoring model (x-axis) versus **BUDE internal energy** (y-axis). Colors indicate generation: purple = first, green = second, yellow = third. Later generations generally converge toward lower energy and greater structural similarity, though outliers exist (e.g., the third repeat for the antiparallel AnP model), which was excluded from subsequent simulations.

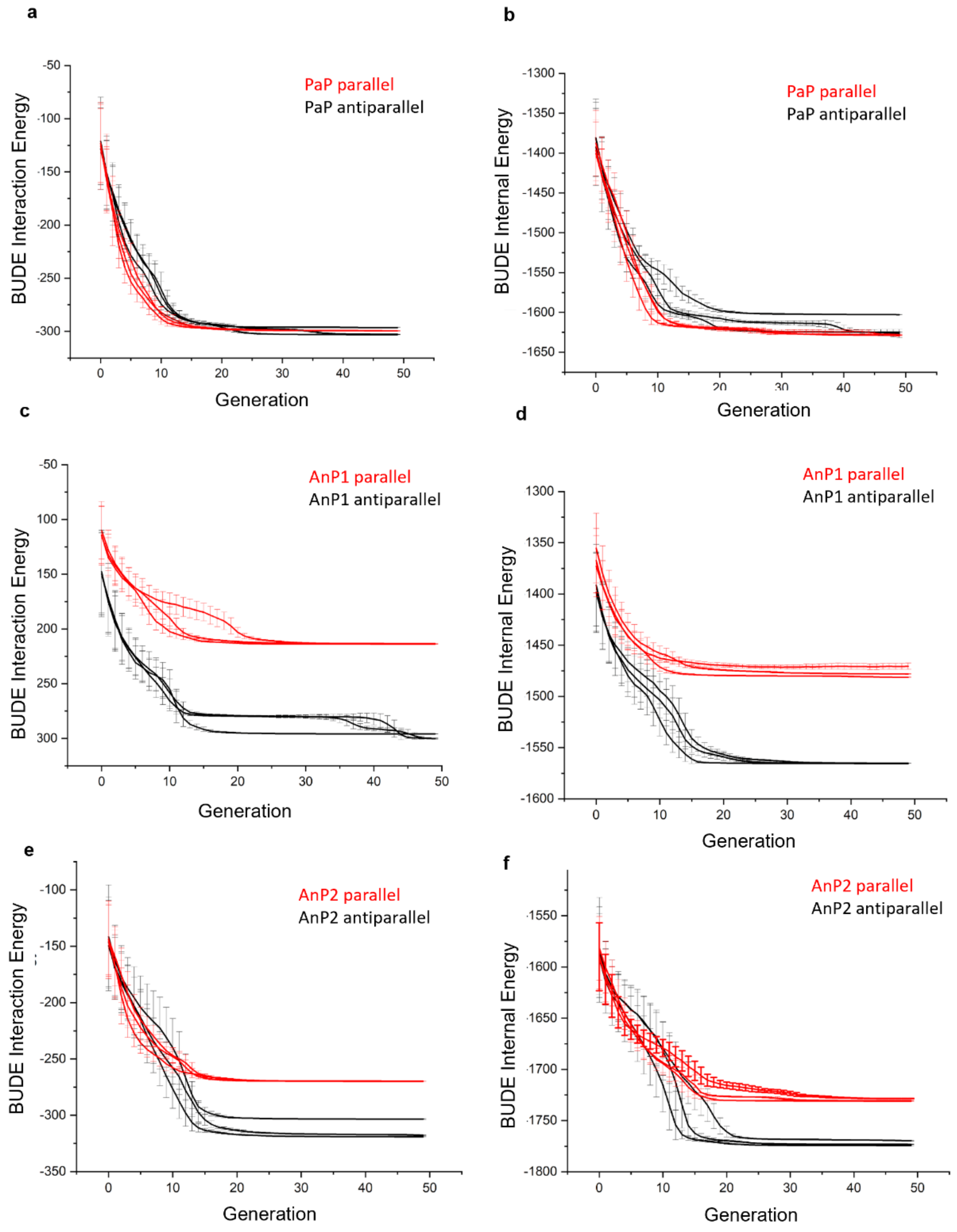

Figure S2-5. Comparison of BUDE energy scores used for coiled-coil parameter optimization in PaP, AnP1 and AnP2. Panels (a, c, e) show interaction energy, and panels (b, d, f) show internal energy. Genetic algorithms were applied to refine parameter sets for both parallel or antiparallel orientations.

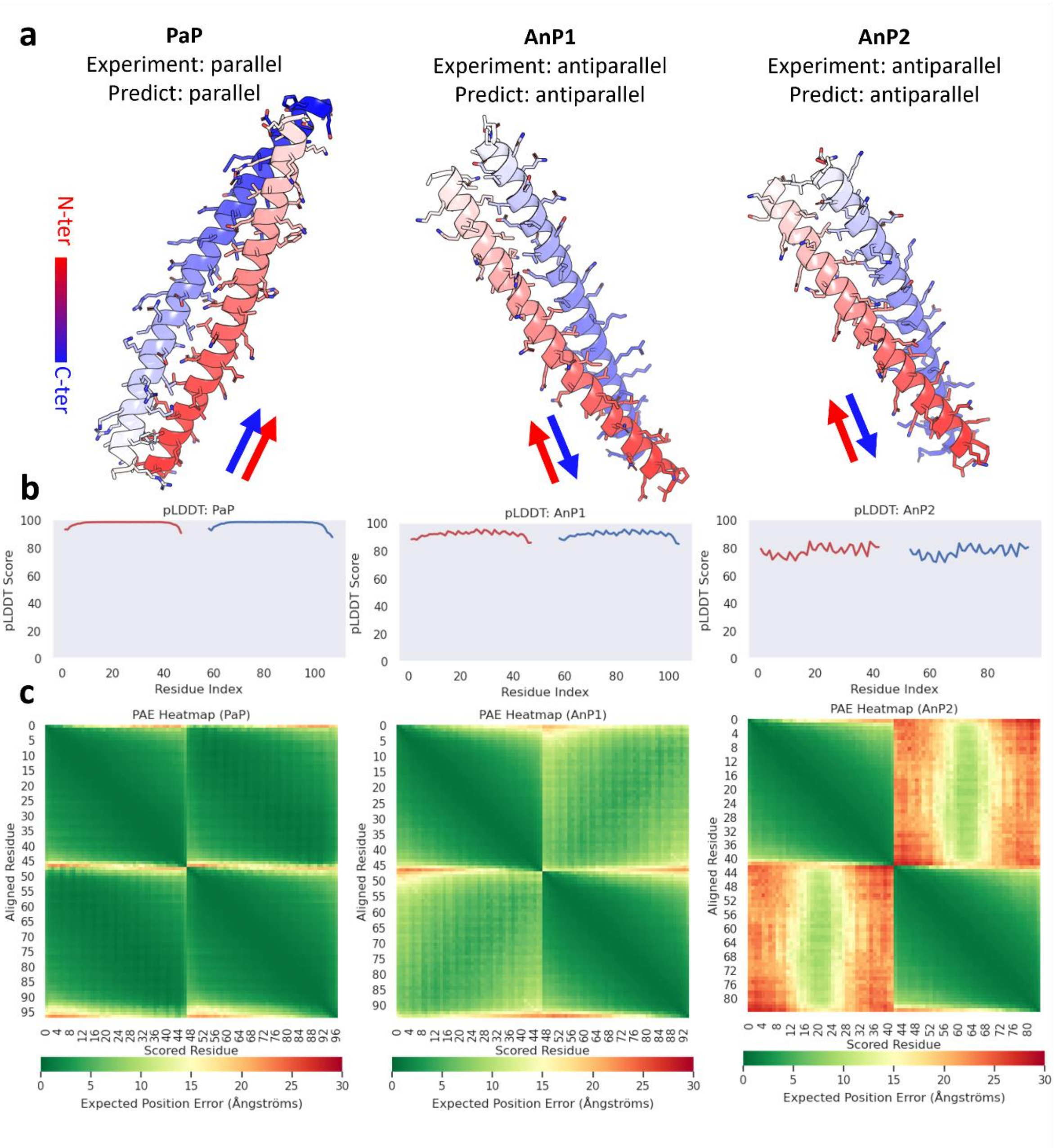

Figure S2-6. The AlphaFold3 predictions for PaP, AnP1, and AnP2 show average pLDDT scores of 97.9, 92.3, and 77.8, respectively (access date: 2024-10-18). Model_3 was selected as the representative structure. (a) shows the predicted coiled-coil structures with N-terminus to C-terminus colored from red to blue. The predicted orientations for PaP, AnP1, and AnP2 align well with experimental data. (b) shows the pLDDT scores across the residue index for each structure. AnP2 has lower pLDDT scores compared to PaP and AnP1, indicating greater uncertainty in the structural prediction. (c) presents the Predicted Aligned Error (PAE) heatmaps for the three structures. The PAE heatmap indicates the confidence in predicting the relative position of residue pairs. AnP2 shows lower PAE confidence compared to PaP and AnP1, highlighting the challenges in accurately modelling its orientation and packing.

SECTION 3 Molecular Simulations

The modelled coiled-coils from AnP1, AnP2 and PaP were then manually joined with the crystal structure of CTA2/CTB (PDB: 1XTC) using PyMOL, and they were then subjected to molecular dynamics simulations. To measure the angle between two CTB units within the same protein complex, we constructed vectors that passed through the centres of two planes (Figure S3-1a). These planes were defined by the positions of the His 57 and Glu 79 residues at the top and bottom of each CTB unit, respectively. Additionally, we calculated the distance between the centroids of the His 57 residues from the two CTB units to define the distance between the two CTBs.

After an initial 100 ns equilibration period, the MD simulations were extended for different durations: 940 ns for PaP, 557 ns for AnP1, and 487 ns for AnP2. We then used this data to create histogram plots (Figure S3-1b), which visually represented the variations in distance and angle for each of the three protein complexes. The analysis revealed that AnP2 was more rigid in terms of distance than AnP1, with AnP1 showing a Gauss fit full width at half maximum (FWHM) of 1.43 ± 0.09 nm compared to 0.69 ± 0.05 nm for AnP2. However, AnP2 displayed the most variation in angle among the three complexes, with an FWHM of 30.32 ± 3.6 degrees.

This difference in rigidity between AnP1 and AnP2 might be due to AnP2's shorter CTA2 peptide (Figure S1-2). Interestingly, our MD simulations of the native CTA2/CTB complex showed that the solvent-exposed N-terminus of the CTA2 peptide is flexible and does not consistently maintain a helical conformation. The helicity of the CTA2 peptide was quantified using the HELO algorithm (Figure S3-2). Along the linker region, the CTA2 helix was divided into overlapping quadruplets, from i to i+3. The x, y, and z coordinates of each residue's Cα atom were used to calculate the three direction cosines (cos ζ) of each quadruplet, and 1.5 Å rise was chosen as the distance between each residue and the helical axis. To calculate the local helicity, 1 – (cos2 ζ1 + cos2 ζ2 + cos2 ζ3) will be used as the criteria, and a non-helical geometry is indicated by a significant deviation of more than 0.2.

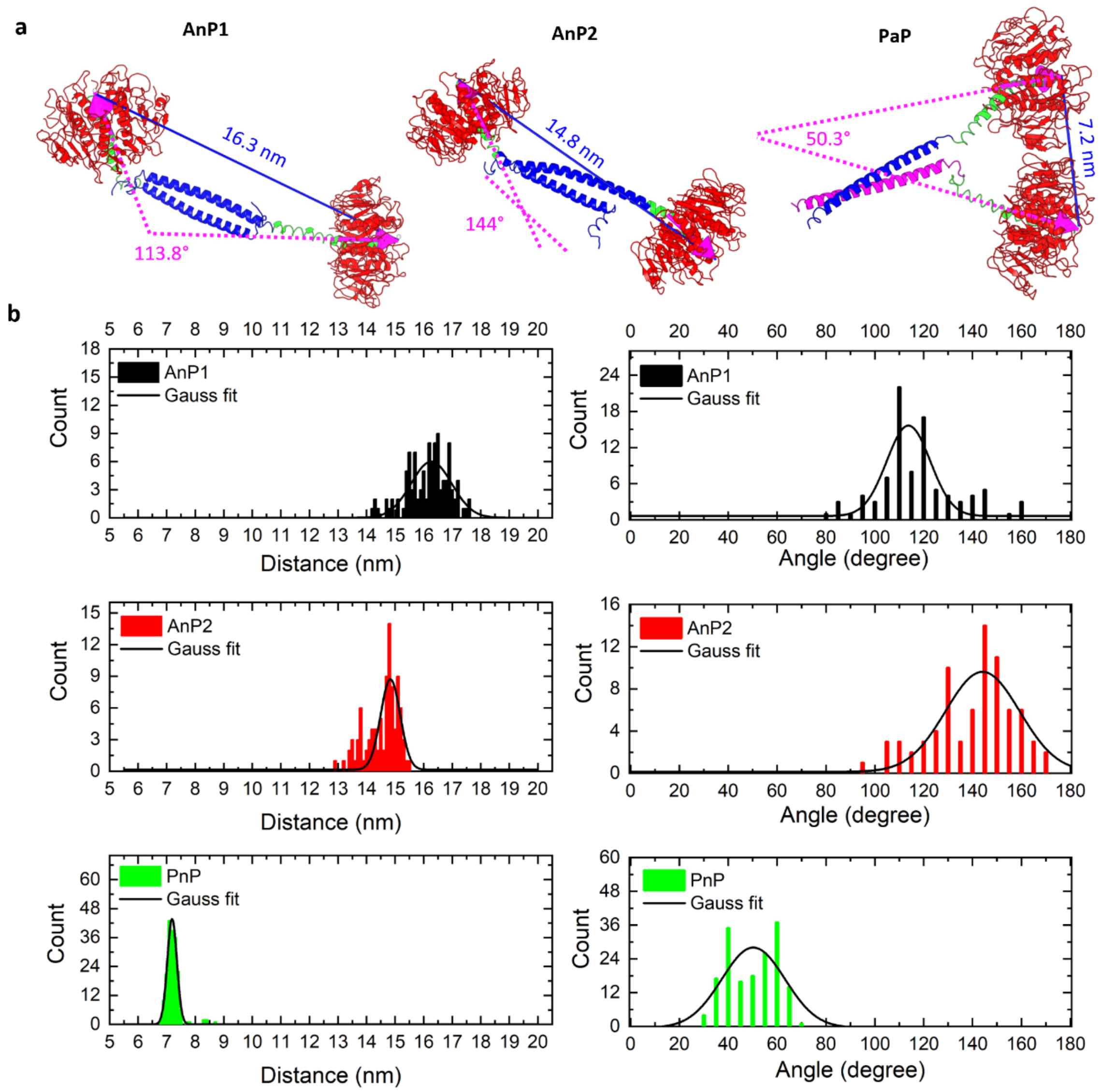

c

	Simulation time	Angle (median)	Angle (FWHM)	Distance (median)	Distance (FWHM)
**AnP1**	557 ns	113.8 ± 1.2	17.9 ± 2.5	16.3 ± 0.0	1.4 ± 0.1
**AnP2**	487 ns	144.1 ± 1.6	30.3 ± 3.6	14.8 ± 0.0	0.7 ± 0.0
**PaP**	940 ns	50.3 ± 1.5	25.9 ± 3.2	7.2 ± 0.0	0.3 ± 0.0

Figure S3-1. (a) Representative models of AnP1, AnP2, and PaP. (b) The Gaussian distributions of distance and angle calculation for the AnP1, AnP2 and PaP complexes modelled during MD simulations. The distance range of 5.5-20 nm was binned into 0.5 nm intervals, and the angle range of 0-180°. (c) Summarise Gaussian fitting data. The table presents the simulation time, median angle and distance with their respective distribution characterised by the Full Width at Half Maximum (FWHM). The FWHM values quantify the dispersion of data around the median, thus providing a measure of variability within each simulation run.

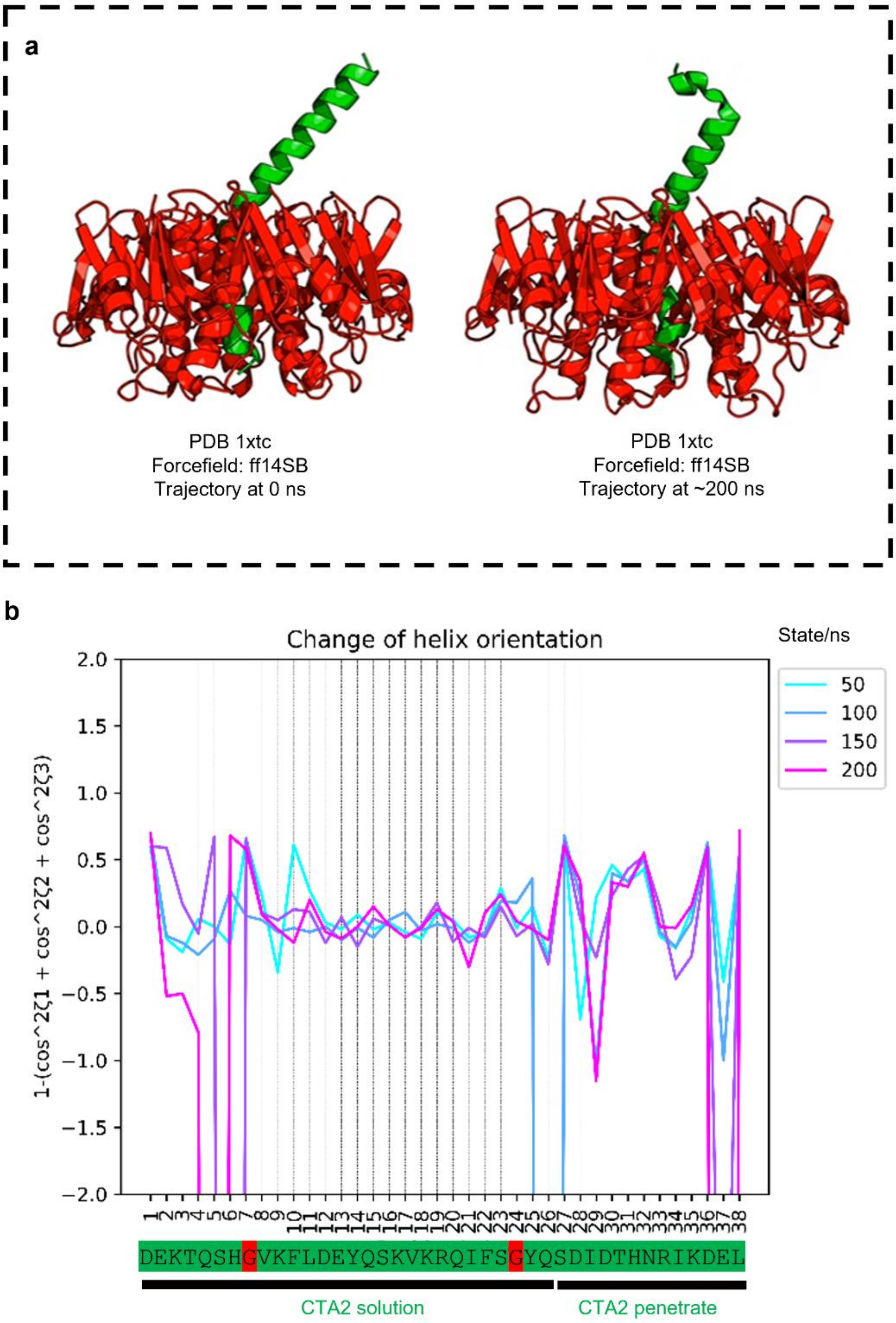

Figure S3-2 Quantitatively assessing the helicity of the CTA2 peptide. (a) The conformations of CTA2/CTB complex are compared at distinct simulation time. The first, marked as "0 ns", is the structure directly sourced from the PDB database (PDB: 1xtc). For this structure, CTA1 and a segment of the CTA2 peptide at the C-terminus were removed. The second, labelled "200ns", represents a structure derived from one trajectory following 200 ns of MD simulation. (b) A plot of 1 – (cos2 ζ1 + cos2 ζ2 + cos2 ζ3) versus the number of residues shows the conformational change at simulation times of 50 ns, 100 ns, 150 ns, and 200 ns. Notably, the two glycine residues located at the 8th and 26th position in the sequence significantly influence the tilt of the part of solution-exposed CTA2 peptide.

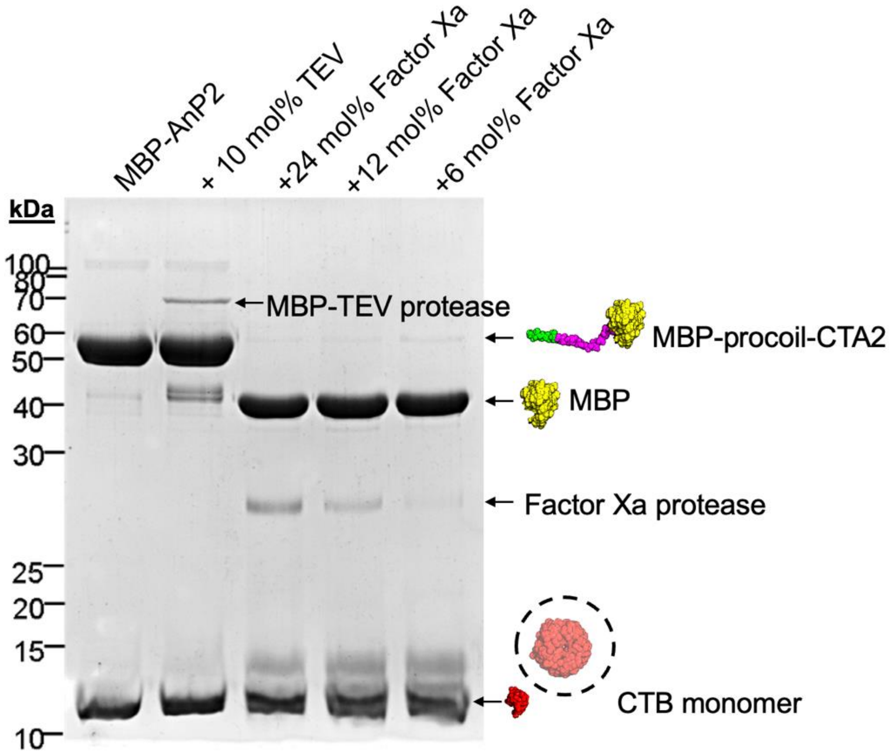

Figure S4-1. Proteolysis of the MBP-AnP2 dimer analyzed by SDS-PAGE (all samples boiled). TEV protease showed minimal cleavage at 10 mol% after overnight incubation at 25 °C, whereas Factor Xa achieved substantially higher cleavage efficiency under similar conditions (6 mol%, 25 °C overnight).

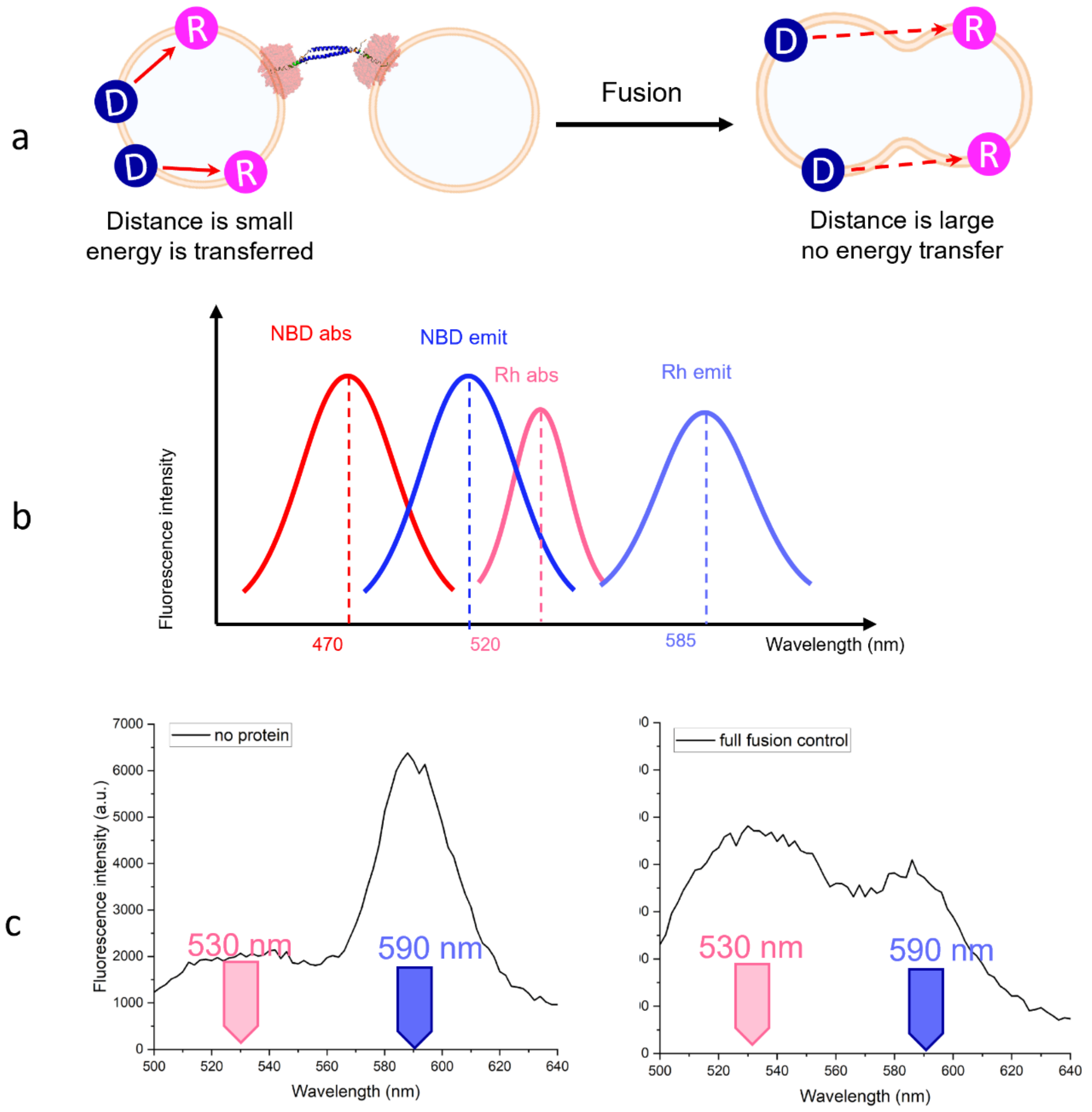

Figure S5-1 (a) Schematic of protein-mediated liposome fusion monitored by proximity-dependent fluorescence resonance energy transfer (FRET). After lipid exchange with probe-free liposomes, the average distance between NBD (Donor) and Rhodamine (Receptor) in FRET-LUV increases. (b) Schematic of NBD and Rhodamine absorbance and emission wavelengths. NBD absorbs at 470 nm and emits at 520 nm, which overlaps rhodamine's absorbance range, enabling rhodamine emission at 585 nm. (c) Fluorescence spectra (500-640 nm) before and after lipid exchange.

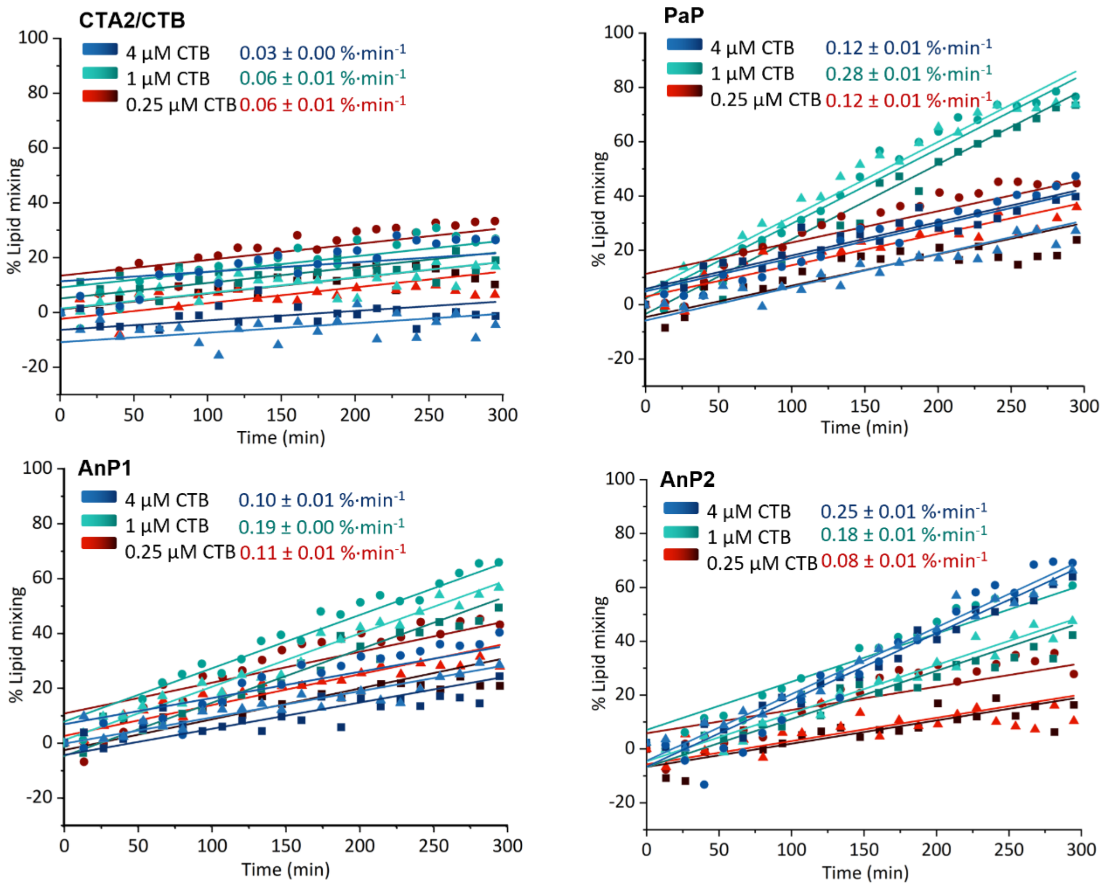

Figure S5-2 Kinetics of lipid mixing at selected concentrations of CTB for different CTA2/CTB constructs. The plot shows the percentage of lipid mixing over time. Each point represents a single independent experiment. As the lipid mixing rate has not begun to plateau within the experimental time window, a global linear regression fit (solid lines) was applied to the three independent replicates for each concentration, yielding the initial lipid mixing rate at each fusogen concentration.

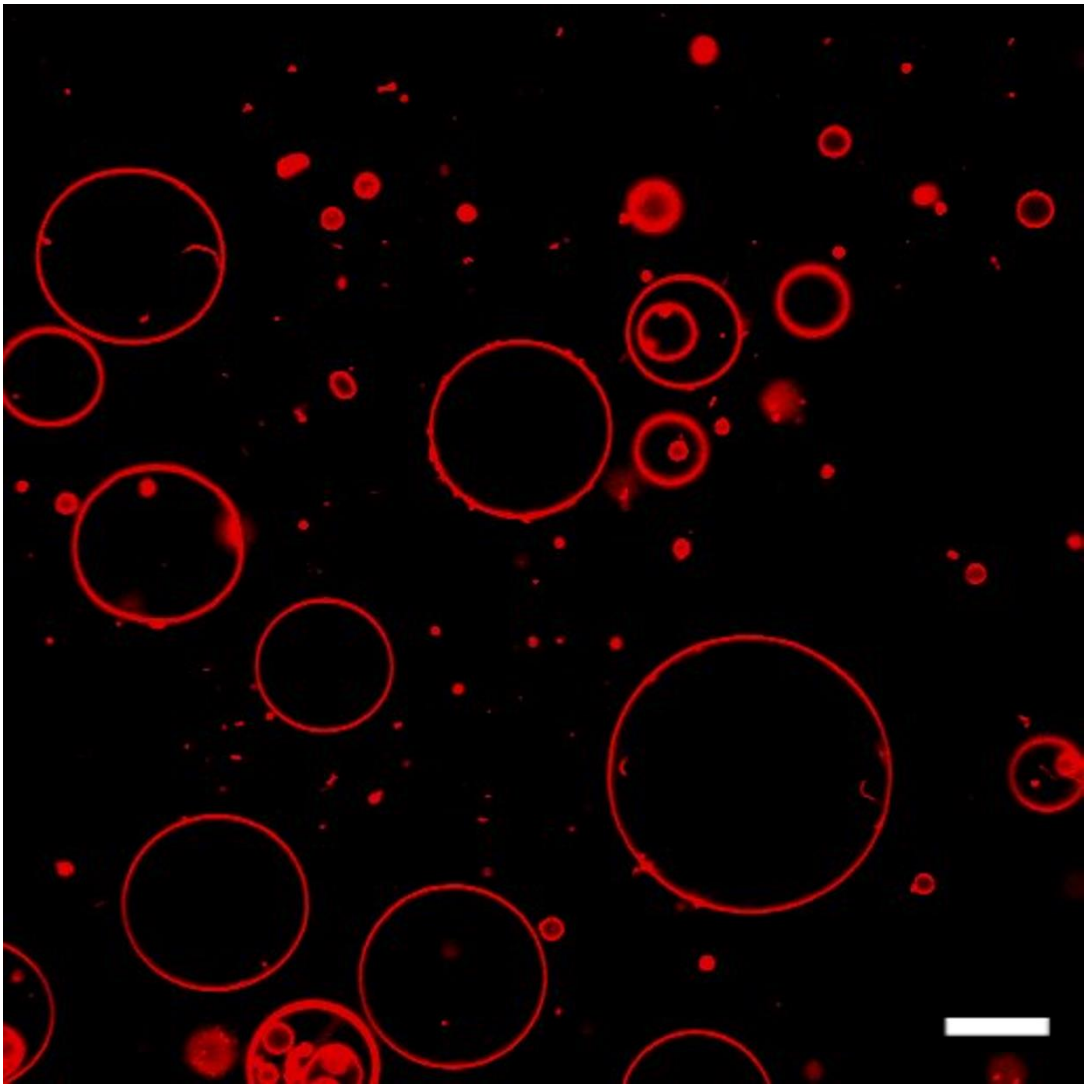

Figure S6-1 Negative control for the GUV experiments employing GUVs comprising of 0.3 mol% Atto647N-labelled lipid, 64.7 mol% DOPC, 30 mol% cholesterol, and 5 mol% GM1, without the any protein present. These GUVs showed no evidence of crosslinking, and therefore no lipid exchange or fusion. Scale bar =10 μm.

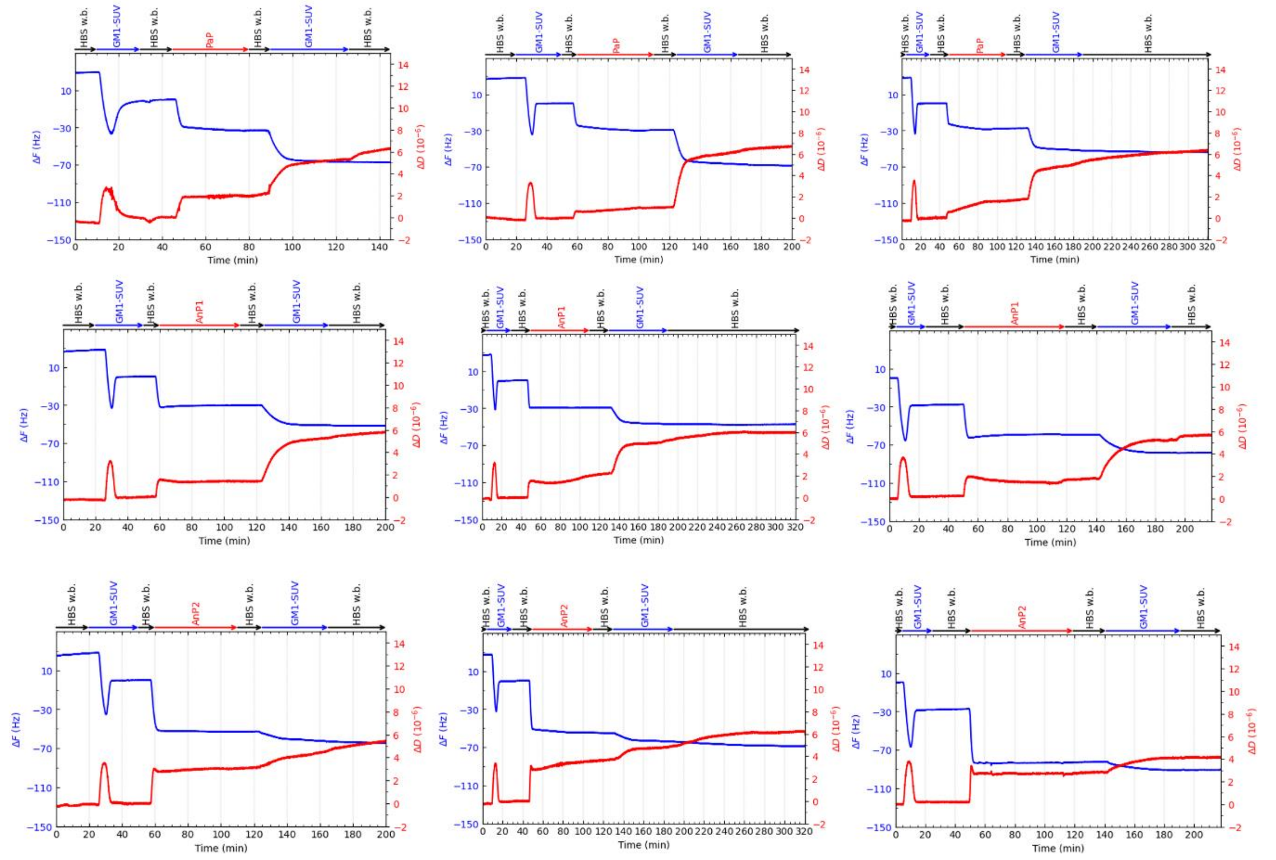

Figure S7-1. QCMD analysis of protein binding to a 5 mol% GM1-coated supported lipid bilayer, followed by addition of a second layer of GM1-SUVs to test for GM1-dependent interactions. Each plot shows three replicates per protein sample (PaP – top; AnP1 – middle; AnP2 - bottom). Arrows indicate the start and duration of incubation steps with either protein samples or HBS working buffer (w.b.). Concentrations:GM1-SUVs – 50 µg/mL; Proteins (PaP – top; AnP1 – middle; AnP2 - bottom) – 200 nM CTB dimer concentration (equivalent to 400 nM CTB); DOPC-SUVs - 50 µg/mL.

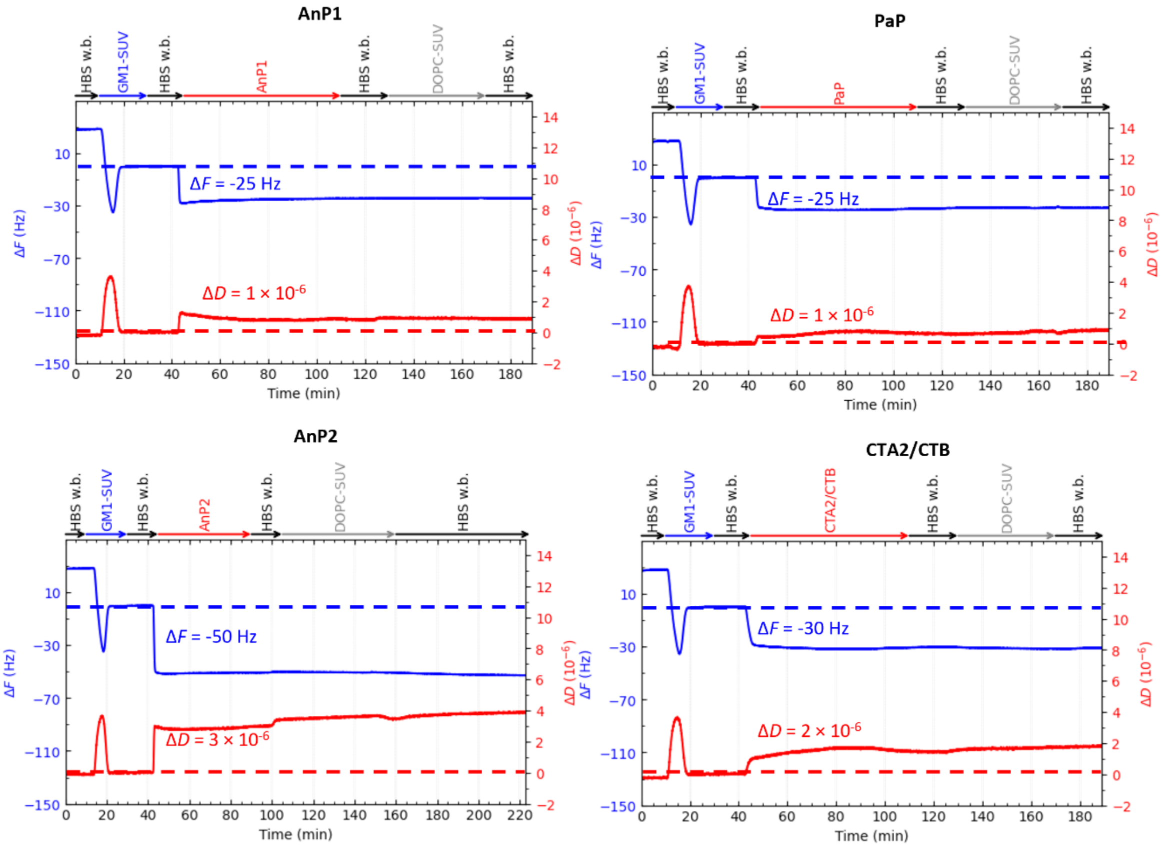

Figure S7-2. QCM-D control data showing that DOPC-SUVs do not bind to the proteins on the sensor (ΔF – blue solid lines; ΔD – red solid lines; overtone $i = 5$; dashed lines in matching color indicate the selected $\Delta F = 0$ and $\Delta D = 0$ baselines). A GM1-SUV bilayer (5 mol% GM1, 95 mol% DOPC) was formed before introducing protein constructs (AnP1, PaP, AnP2, or CTA2/CTB). Passing DOPC-SUVs over the protein-coated surface caused no additional ΔF change, confirming lack of binding. Data shown are representative of three replicates per protein. Arrows above each graph indicate the start and duration of incubation steps with samples or HBS working buffer (w.b.). Concentrations: GM1-SUVs – 50 µg/mL; Proteins (AnP1, AnP2, PaP, or CTA2/CTB) – 200 nM CTB dimer concentration (equivalent to 400 nM CTB) ; DOPC-SUVs - 50 µg/mL.

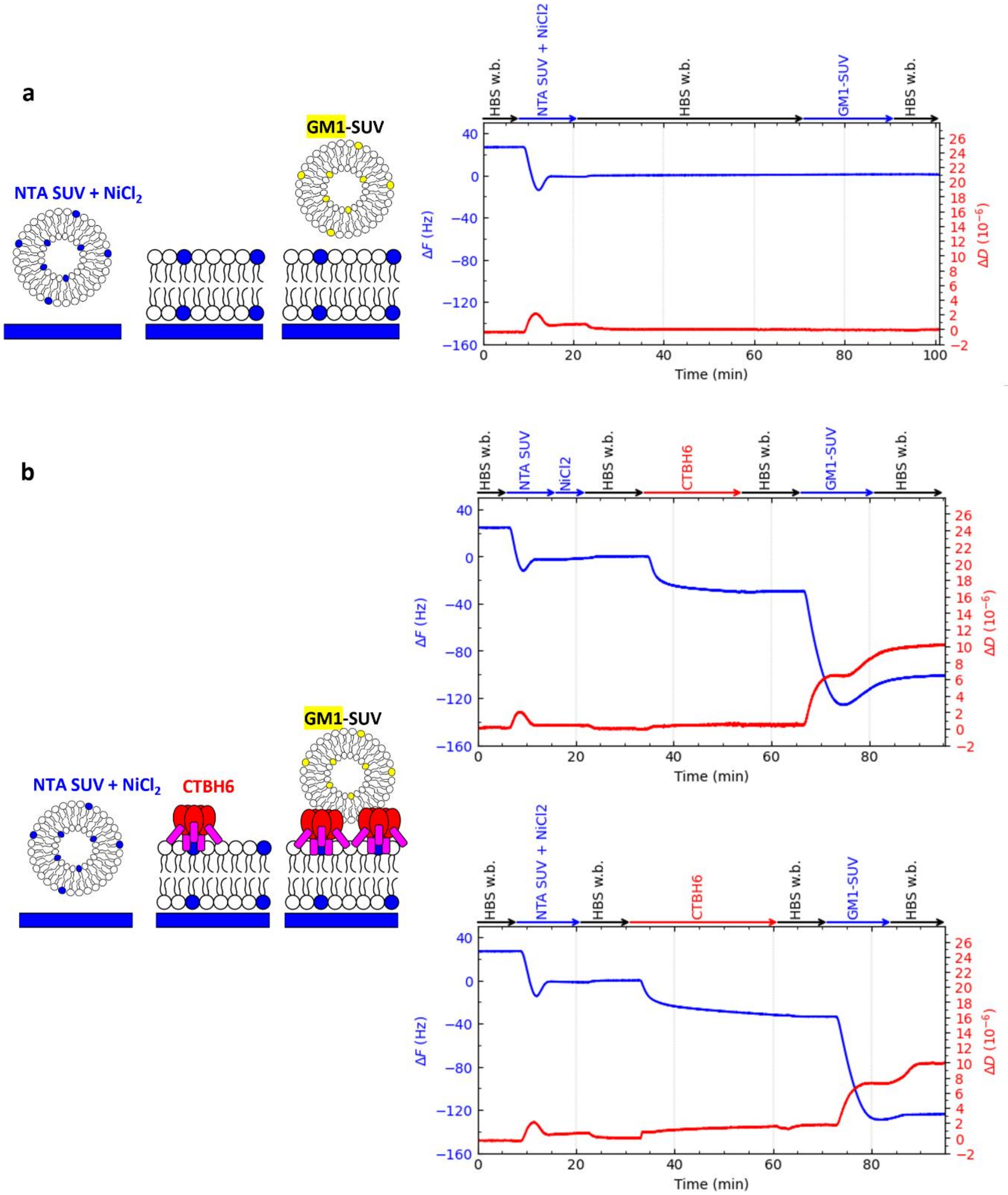

Figure S7-3 QCM-D analysis of GM1-SUV binding to CTB-H6-functionalized supported lipid bilayers. CTB-H6 carries a C-terminal hexahistidine tag on the face opposite the GM1-binding sites,[6] so when attached to a Ni-NTA surface, GM1-binding sites face outward into solution. (a) **Control**: sequential assembly of a Ni-NTA-DOPC SLB, followed by incubation with GM1-SUVs in the absence of protein. No significant ΔF or ΔD changes confirm GM1-SUVs do not bind non-specifically to Ni-NTA SLBs. (b) **CTB-H6-functionalized SLB** (in duplicate)**:** After Ni-NTA SLBs were incubated with CTB-H6, GM1-SUV injection caused large ($\Delta F \approx -90$ Hz; $\Delta D \approx 8 \times 10^{-6}$) indicating GM1-SUVs binding. Arrows above the graph indicate the start and duration of incubation steps with samples or HBS working buffer (w.b.). Concentrations: GM1-SUVs – 50 μg/mL; Proteins (CTB-H6) – 400 nM; NTA SUV - 50 μg/mL.

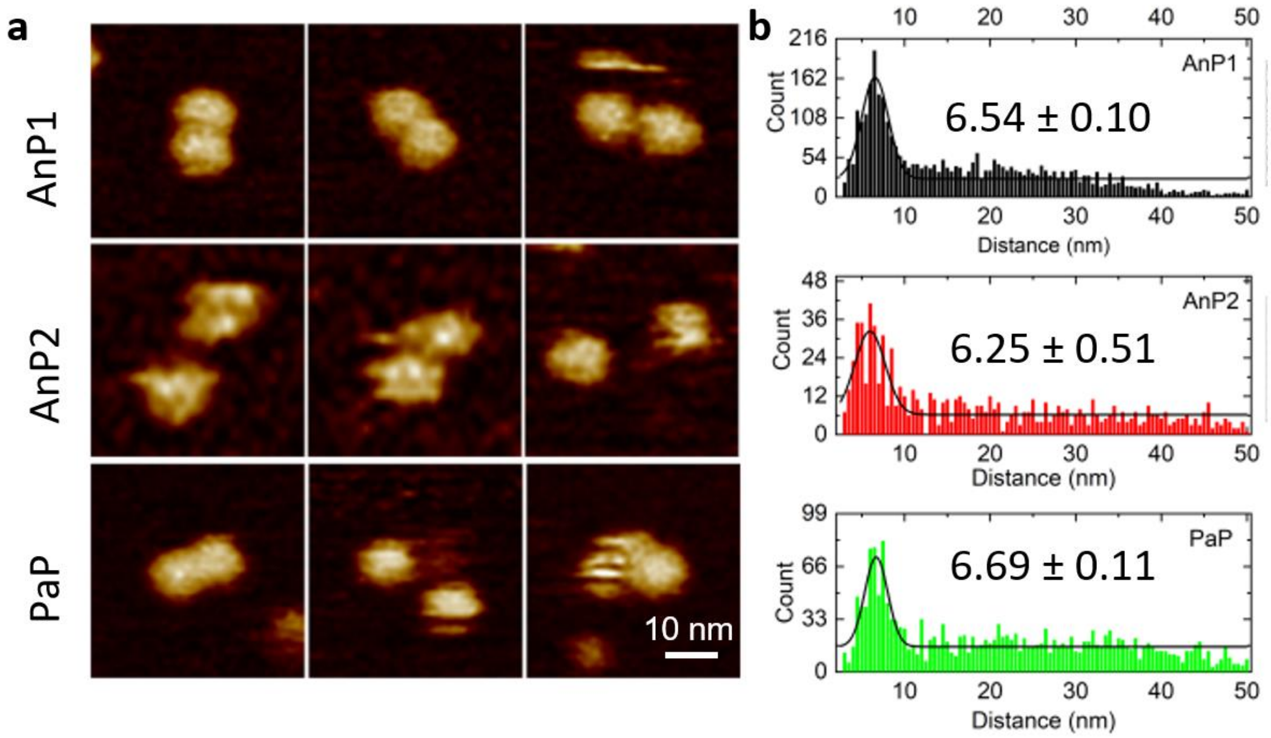

Figure S8-1 AFM imaging of AnP1, AnP2, and PaP bound to nickel-treated mica surfaces. (a) Representative AFM images of each protein complex adsorbed to the surface. (b) Frequency distribution of distances between surface-bound particles of the three proteins. The histogram for the calculation covered a distance range of 2.5 nm to 50 nm, with a bin width of 2.5 nm. All three complexes showed most frequent distances of 6.25–6.69 nm, consistent with the ~6 nm width of the CTB pentamer, indicating a flat surface orientation.

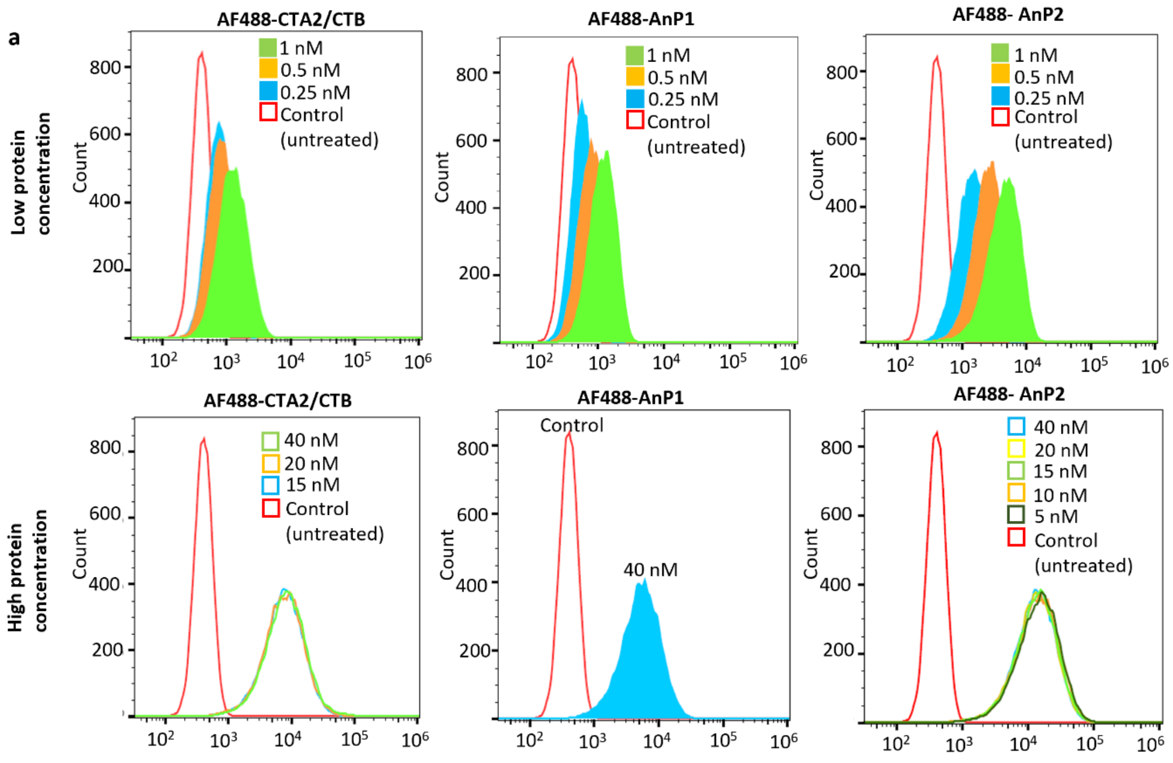

Figure S9-1 Flow cytometry histograms showing binding of Alexa Fluor 488 (AF488)-labelled CTB complexes to Jurkat cells at varying concentrations. Upper panels represent low CTB concentrations; lower panels represent high concentrations. Untreated cells served as controls.

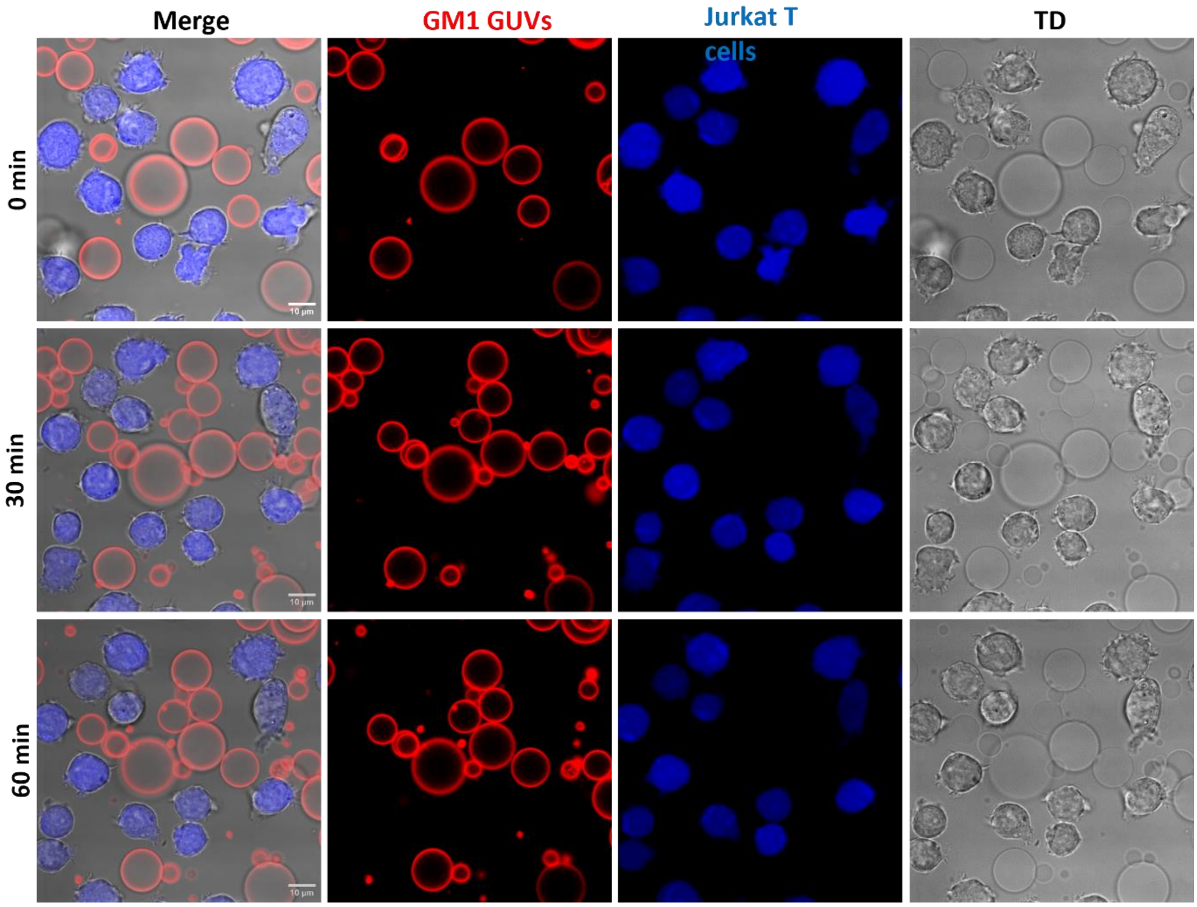

Figure S10-1 Confocal microscopy images of 5 mol% GM1-decorated GUVs and Jurkat T cells incubated without CTB. GUVs (red) were labelled with the fluorescent lipid DOPE-Atto647N; Jurkat cells (blue) were stained with CellTrace™ Violet. Live-cell imaging was performed at 37 °C using confocal laser scanning microscopy over 60 minutes. TD stands for transmission detector. Scale bar =10 µm. Image represents a single experiment.

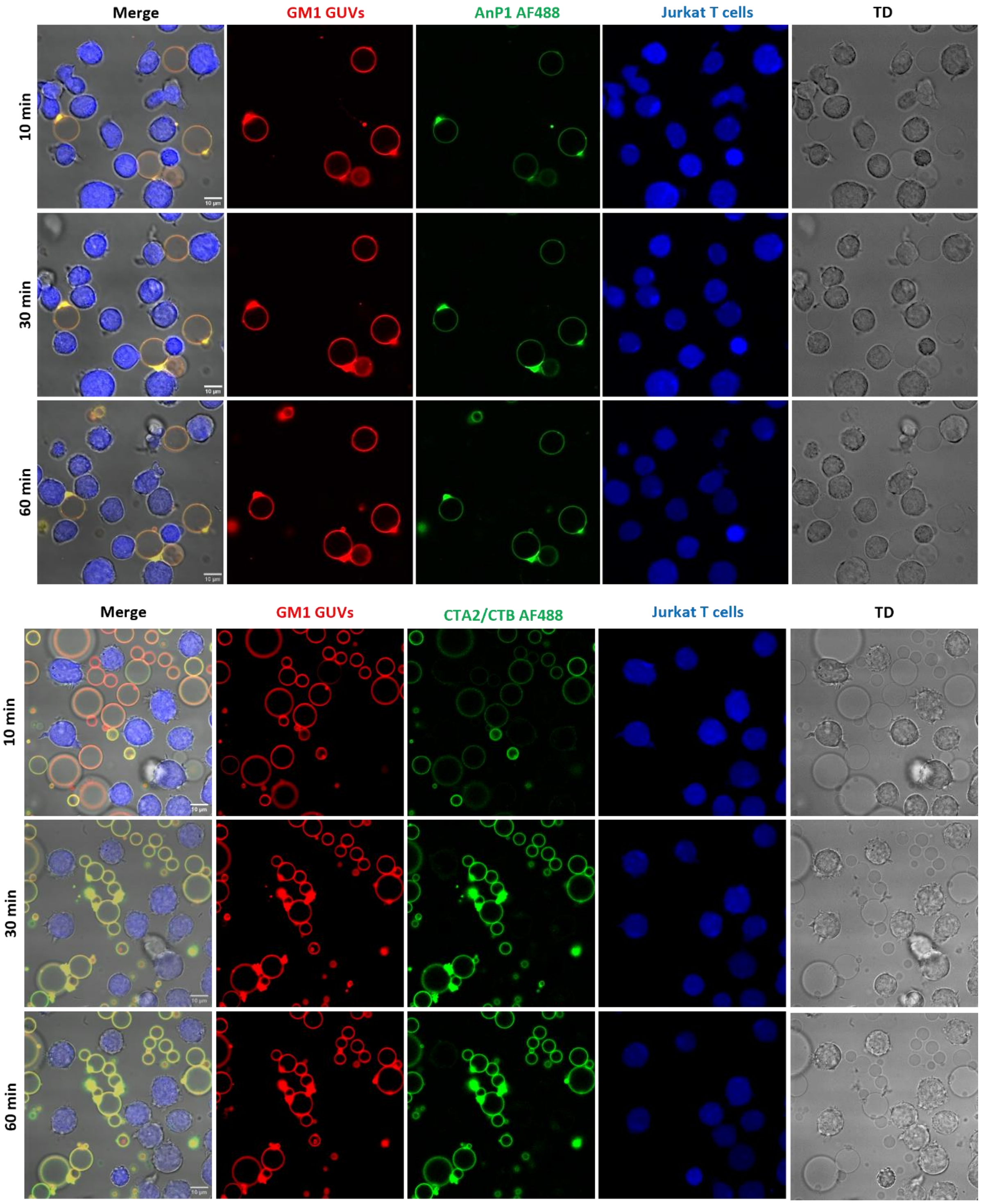

Figure S10-2 Crosslinking of GM1-decorated GUVs and Jurkat T cells mediated by AF488-labeled AnP1 (top panels) or CTA/CTB (bottom panels). In each case the CTB pentamer concentration was 400 nM, and confocal live-cell imaging was conducted at 37 °C for 60 min after protein addition. GUVs (red, DOPE-Atto647N), Jurkat cells (blue, CellTrace™ Violet), and AnP1 or CTA2/CTB (green, AF488) are shown. Scale bars = 10 μm. Images represent one example from four independent repeats.

1. Gradišar, H.; Jerala, R., De Novo Design of Orthogonal Peptide Pairs Forming Parallel Coiled-Coil Heterodimers. *J. Peptide Sci.* **2011,** *17*, 100-106.
2. Thompson, K. E.; Bashor, C. J.; Lim, W. A.; Keating, A. E., Synzip Protein Interaction Toolbox: In Vitro and in Vivo Specifications of Heterospecific Coiled-Coil Interaction Domains. *ACS Syn. Biol.* **2012,** *1*, 118-129.
3. McClain, D. L.; Woods, H. L.; Oakley, M. G., Design and Characterization of a Heterodimeric Coiled Coil That Forms Exclusively with an Antiparallel Relative Helix Orientation. *J. Am. Chem. Soc.* **2001,** *123*, 3151-3152.
4. Gurnon, D. G.; Whitaker, J. A.; Oakley, M. G., Design and Characterization of a Homodimeric Antiparallel Coiled Coil. *J. Am. Chem. Soc.* **2003,** *125*, 7518-7519.
5. Patterson, D. P.; Su, M.; Franzmann, T. M.; Sciore, A.; Skiniotis, G.; Marsh, E. N. G., Characterization of a Highly Flexible Self-Assembling Protein System Designed to Form Nanocages. *Protein Sci.* **2014,** *23*, 190-199.
6. Arnott, Z. L. P.; Morgan, H. E.; Hollingsworth, K.; Stevenson, C. M. E.; Collins, L. J.; Tamasanu, A.; Machin, D. C.; Dolan, J. P.; Kamiński, T. P.; Wildsmith, G. C.; Williamson, D. J.; Pickles, I. B.; Warriner, S. L.; Turnbull, W. B.; Webb, M. E., Quantitative N- or C-Terminal Labelling of Proteins with Unactivated Peptides by Use of Sortases and a D-Aminopeptidase. *Angew. Chem. Int. Ed.* **2024,** *63*, e202310862.
7. Wood, C. W.; Heal, J. W.; Thomson, A. R.; Bartlett, G. J.; Ibarra, A. Á.; Brady, R. L.; Sessions, R. B.; Woolfson, D. N., Isambard: An Open-Source Computational Environment for Biomolecular Analysis, Modelling and Design. *Bioinformatics* **2017,** *33*, 3043-3050.
8. Rhys, G. G.; Wood, C. W.; Lang, E. J. M.; Mulholland, A. J.; Brady, R. L.; Thomson, A. R.; Woolfson, D. N., Maintaining and Breaking Symmetry in Homomeric Coiled-Coil Assemblies. *Nature Commun.* **2018,** *9*, 4132.
